# PROTOCOL: Water, sanitation and hygiene for reducing childhood mortality in low‐ and middle‐income countries

**DOI:** 10.1002/cl2.1135

**Published:** 2021-01-19

**Authors:** Hugh Sharma Waddington, Sandy Cairncross

**Affiliations:** ^1^ London School of Hygiene and Tropical Medicine London International Development Centre London UK; ^2^ London School of Hygiene and Tropical Medicine London UK

## Abstract

Respiratory tract infections and diarrhoea are the two biggest killers of children in low income contexts. They are closely related to access to, and use of improved water, sanitation and hygiene (WASH). However, there is no high quality systematic review that quantifies the effect of WASH improvements on childhood mortality. Existing systematic reviews of WASH improvements measure effects on morbidity, under the (often implicit) assumption that morbidity is closely correlated with mortality. This is at least partly because the impact evaluations on which they are based are only designed to detect changes in morbidity with statistical precision, whereas mortality is a relatively rare outcome. The proposed review will address this evidence synthesis gap, using the greater statistical power of meta‐analysis to pool findings across studies.

## 1 BACKGROUND

### 1.1 One thousand children died today because of diarrhoea

Water, sanitation and hygiene (WASH) are fundamentally important for human life, health and happiness. Maslow (1943) proposed a hierarchy of goals for human life in the following order: “physiological, safety, love, esteem, and self‐actualization” basic needs. The physiological needs relate to healthy regulation of the human body's metabolism via sufficient access to air, water, nutrition, warmth, rest (including sleep) and the means to excrete. Safety was placed just above physiological needs, and linked specifically to safety from illness and pain in childhood, as well as from “wild animals” and “assault” throughout the life‐course. It is quite difficult to over‐emphasise the contribution of sufficient water, sanitation and hygiene to ensuring basic needs are met.

Yet, according to the Joint Monitoring Programme (JMP), two billion people do not have safe, readily available water at home, and 4.5 billion lack access to safely managed sanitation services (WHO/UNICEF, 2019). In sub‐Saharan Africa, 400 million people have to use surface water or improved water sources that take more than 30 min to reach, queue for and return home. Of the 1.4 billion people worldwide who defecate in the open or use unimproved or shared sanitation facilities, half a billion live in South Asia (around 400 million in India) and another half billion are in sub‐Saharan Africa.

Inadequate WASH can contribute to the outbreak and chronic presence of preventable infections like pneumonia and diarrhoeal disease, which are the two biggest killers of children globally (Liu et al., 2012).^1^ Enteric disease may also cause tropical enteropathy, a subclinical disorder where the lining of the gut wall is damaged by repeated bouts of infection until it is unable to absorb nutrients adequately (Humphreys, 2009; Shiffman et al., 1978). Chronic high enteric infection rates are among the leading causes of undernutrition and death in children in developing countries (Cairncross et al., 2014).

**Figure 1:**
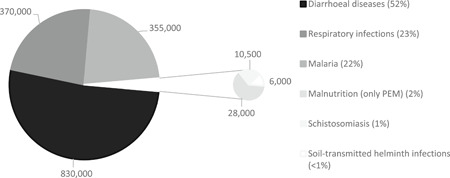
Estimated annual global deaths due to inadequate WASH. PEM protein energy management. *Source*: Chirgwin et al. (submitted).

Water‐related diseases are responsible for an estimated 21% of the global disease burden (Black et al., 2010). According to recent Global Burden of Disease (GBD) estimates (Prüss‐Ustün et al., 2019), inadequate WASH is associated with 1.6 million deaths per year, due to diarrhoea, acute respiratory infection, malnutrition due to protein energy management and, as a result of water mismanagement, malaria (Figure 1). Diarrhoea alone kills 850,000 people every year, 300,000 of whom are children aged under 5 (Prüss‐Ustün et al., 2019). This is equivalent to a line of dead children from London to Cardiff every year, every death being a personal tragedy (White, 2004).

Parasitic worm infections, associated with inadequate sanitation (e.g., Ascaris, Trichuris and hookworm infections), are responsible for 39 million disability‐adjusted life years (DALYs), equivalent to the global burden of mortality for malaria and tuberculosis combined (Stephenson et al., 2000; see also Ziegelbauer et al., 2012 frisk of). Trachoma, a water‐washed eye infection causing blindness, spread by the *Musca sorbens* fly which breeds in human excrement, affects an estimated 146 million people worldwide (Freeman et al., 2017; Rabiu et al., 2012). Water supply changes may also affect rates of arsenic poisoning due to groundwater consumption, which can cause nutritional deficiency, cancer and death (Dar & Khan, 2011; Jones‐Hughes et al., 2013).

There may also be important externalities from private consumption of improved WASH services through environmental health spillovers (Barreto et al., 2007; Duflo et al., 2015; Root, 2001; Spears, 2013). These operate in the private domain (household and yard) and public domains (places of work, education, commerce, recreation, street and fields) (Cairncross et al., 1996). For example, the World Bank (2008) estimated environmental costs of poor sanitation at 2% of GDP in South Asia (Cambodia, Indonesia, the Philippines and Vietnam). Poor access and use of WASH in places with high population density, may explain why some countries, particularly in South Asia, have worse child malnutrition outcomes than their income levels alone would predict (Spears, 2013).

While all suffer loss of dignity from open defecation and drudgery from water collection, women and girls suffer particularly. Women do the majority of water carrying when households lack access to an improved water source in Africa and Asia (Sorenson et al., 2011). Originally, McSweeney (1979) had reported that the burden of time spent on domestic chores in Burkina Faso started in a girl's childhood, was around 7–8 h/day by age 9 (double that of boys of similar age) and women and girls were responsible for all of the water collection. Feachem et al. (1978) estimated that 96% of water collections in Lesotho were made by women and girls. Cairncross and Cliff (1987) reported time savings associated with water supply improvements for women in Mozambique, which were put to other household activities (food preparation and childcare), suggesting a possible mechanism through which WASH impacts on nutrition, and therefore possibly child survival (see also Dangour et al., 2013). Women and girls still do most water collection in 24 sub‐Saharan Africa countries (Graham et al., 2016), risking becoming pedestrian road casualties, and risking attack and assault by “pests and perverts” (Campbell et al., 2015). For example, Cairncross and Cliff (1987) found in northern Mozambique that, when the functioning village standpipe broke down, women were forced to rely on traditional sources. The choice included a water source 8 km away, taking between 4 and 7 h (travel time and queueing) for the return journey, or one 4 km away, where “[a] few women spent the night… despite the danger of lions, waiting for water to appear in the holes dug for that purpose” (p. 51).

Women and girls can also be put in danger when they have to wait until after dark to urinate or defecate with privacy (Sommer et al., 2014; Sorenson et al., 2011). For example, studies in Kenya (Winter & Barchi, 2016) and India (Jadhav et al., 2016) found that women who openly defaecated were more likely to experience nonpartner sexual and/or physical violence; in India, which compared women who openly defaecated with those with a private toilet, the difference was 200%. There may also be adverse maternal and child health implications due to inadequate WASH services in health facilities and other places of newborn delivery (Benova et al., 2014). Pregnant women and neonates are thought to be a particularly high‐risk group because infection and sepsis are major causes of maternal and neonatal mortality (Liu et al., 2012). More generally, disadvantaged groups, such as children, the elderly, women, poor people, immunocompromised people such as those living with human immunodeficiency virus (HIV) and people with disabilities, are less likely to have access to appropriate WASH technologies (whether drinking water supplies of sufficient quantity and quality, means of safe excreta disposal and hygiene practices), and therefore more likely to experience mortality and negative health and socioeconomic consequences.

Where female adults are required to collect the water, which is the majority of cases, older children may be pulled out of the school to care for younger ones (Koolwal & van de Walle, 2010). Diminished educational attainment, due to children's school enrolment and attendance as well as teacher attendance, as well as delayed entry to the labour market, have implications for employment, life‐time wage earnings and poverty (Hutton et al., 2007; Poulos et al., 2006).

Other longer‐term economic implications arise due to delayed entry to the labour market, and monetary losses due to costs of medical treatment and aversion costs of treating and storing unclean water or purchasing water from vendors (Bosch et al., 2002; Cairncross & Kinnear, 1992). These costs can be exorbitant for poor households in urban informal settlements (slums) who are unserved by house connections. For example, the costs of vendor supply were estimated at 7–11 times higher than public utility water supply in Nairobi, Kenya, 12–25 times in Dacca, Bangladesh, 28–83 times higher in Karachi, Pakistan, 17–100 times higher in Port‐au‐Prince, Haiti, and 100 times higher in Nouakchott, Mauritania (Bhatia & Falkenmark, 1993, p. 14). In a study in Khartoum, Sudan, where up to 56% of household income in squatter areas was spent on vendor water (Cairncross & Kinnear, 1992), the income and price elasticities of demand for water were found to be very inelastic (that is, demand is relatively unresponsive to changes in income and price). It was therefore suspected that the poorest households would need to substitute food expenditure to meet water needs, causing malnutrition.

For all of these reasons, inadequate WASH service access and use is likely to support vicious cycles of limited human development and weak economic growth (Ramirez et al., 1998). It is very important, therefore, to understand the likely magnitude of the impacts of WASH interventions on important outcomes, such as mortality, in particular contexts and for particular groups.

### 1.2 Water, sanitation and hygiene interventions

WASH interventions have several important components to them (Chirgwin et al., submitted) including: the technology that is provided to users (e.g., a child's potty and knowledge about safe excreta disposal); the intervention mechanism used to encourage demand among the target population (e.g., a government subsidy on the potty purchase price and promotional campaign about excreta disposal) or to improve supply (e.g., capacity building for sanitation providers); and the social and physical environment where participants use the technology (e.g., the household and yard).^2^

#### 1.2.1 WASH technologies

The quality of water supply, sanitation and hygiene facilities—that is, the extent to which they are likely to provide drinking water of sufficient quantities for basic needs, enable hygienic hand‐washing and food preparation, and safe removal of excrement from the human environment—is dependent on the type of technology. These are usually grouped into drinking water, sanitation and hygiene ladders (Table 1).^3^

**Table 1:** Water, sanitation and hygiene technology ladders

	Drinking water	Sanitation	Hygiene
Improved facilities: safely managed	Improved facilities that:	Improved facilities where waste products are either:	Undefined
	are accessible on premises, and provide water when needed, and provide water free from contamination.		
		treated and disposed in situ, or temporarily stored and then emptied and transported to off‐site treatment centre, or transported through sewer with wastewater and treated off‐site	
Improved facilities: basic	Improved sources that require <30 min round‐trip to collect (including queueing time). These include piped supplies:	Improved facilities provided at the household level. These include networked sanitation:	Fixed or mobile handwashing facilities with soap and water:
			handwashing facilities defined as a sink with tap water, buckets with taps, tippy‐taps and jugs or basins designated for handwashing soap includes bar soap, liquid soap, powder detergent and soapy water
		flush and pour flush toilets connected to sewers And on‐site sanitation: flush or pour flush toilets connected to septic tanks or pits pit latrines with slabs composting toilets, including twin pit latrines and container‐based systems	
	tap water in the dwelling, yard, or plot public standposts/pipes And nonpiped supplies: boreholes/tubewells protected wells and springs rainwater packaged water, including bottled water and sachet water delivered water, including trucks and small carts		
Limited facilities	Improved sources of the above types requiring more than 30 min to collect including queueing time	Improved facilities of the above types shared by two or more households	Handwashing facilities without soap and water (e.g., ash, soil, sand or other handwashing agent)
Unimproved facilities	Nonpiped supplies:	On‐site sanitation or shared facilities of the following types:	Undefined
	unprotected wells and springs		
		pit latrines without slabs hanging latrines bucket latrines	
No facilities	Surface water (e.g., drinking water directly from a river, pond, canal, or stream)	Open defecation (disposal of human faeces in open spaces or with solid waste)	No handwashing facility on premises

*Source*: WHO/UNICEF (2019); https://washdata.org/monitoring.

#### 1.2.2 Intervention mechanisms

Mechanisms for providing WASH technologies can be categorised into demand and supply side interventions. Demand side interventions include: behaviour change communication, such as health education and psychosocial “triggering,” for example, social marketing and community‐led total sanitation (CLTS); subsidies and microloans for consumers; and legal measures proscribing open defaecation, discharge of contaminated water, or dumping of waste (e.g., Cairncross, 1992). For example, psychosocial triggering aims to promote demand for WASH technology among consumers using directive or participatory methods (De Buck et al., 2017). An example of a directive approach is social marketing, which motivates social change through a combination of product (technology used to meet a need), promotion (to increase desirability and acceptability), place (installation in an appropriate place for users) and price (the cost for users takes into account affordability) (Cairncross, 2004; Evans et al., 2014). These are often implemented at community level such as in schools and health facilities via approaches such as community health clubs to promote demand (Waterkeyn & Cairncross, 2005). Participatory, bottom‐up approaches are also being rapidly scaled up, including CLTS. In CLTS, the community is facilitated to discuss how they would like sanitation practices to change, identify problem areas (e.g., “walks of shame”), and use social cohesion and pressure to motivate people to construct latrines and stop practising open defecation (Kar & Chambers, 2008).

On the supply side, interventions include: direct provision of technology by an external body (e.g., government, NGO); improving operator performance (e.g., institutional reform, capacity building, operator financing, regulation and accountability); privatisation (e.g., Galiani et al., 2005) and nationalisation of service delivery; and promoting small‐scale independent provider involvement (e.g., sanitation marketing through microloans and capacity building for providers) (Poulos et al., 2006). WASH technology may be for use in private (household and yard) or public spaces (shared facilities, WASH in health facilities and schools, places of transit, work, commerce, reaction, streets and fields). Measures to improve service provider performance include measures such as enacting and implementing water quality standards (Cairncross et al., 1996), government regulation of private utility providers, and reforms to operator financing (e.g., payment‐by‐results) (Poulos et al., 2006). Encouraging small‐scale independent providers like nonprofits and the private sector (Sansom et al., 2003) may include microloans for WASH service providers and capacity building. As an example of the latter, sanitation marketing aims to increase availability of sanitation technology and maintenance services (such as pit emptying), by training local artisans to produce sanitation products that are suitable for the varying needs of consumers (e.g., Cameron et al., 2013).

Decentralisation, where community representatives are placed in planning, design, implementation and operation of the WASH service provider, is an example of an intervention category that combines supply and demand. For example, community‐driven development (CDD) uses a bottom‐up approach, block grants with cost sharing and often a component of local institutional strengthening (White et al., 2018).

#### 1.2.3 Place of use

The social and physical environment where participants interact with WASH technology is important for understanding infectious disease transmission. As noted, Cairncross et al. (1996) distinguished private domain (dwelling and yard) and public domains (community, schools, places of work, commerce and recreation, fields in rural areas and streets in cities) in disease transmission. The importance of the differentiation is in the potential for communicable disease transmission—the greater potential for single cases to cause epidemics in public spaces—and the types of interventions that are needed to combat transmission—the greater focus on infrastructure investment and regulation in public space, and personal hygiene in private spaces (which also depends on infrastructure investment especially water supply).

### 1.3 The effects that WASH access and use can have

Outcomes of WASH sector interventions can be categorised into five groups: intermediate outcomes relating to WASH access, knowledge, attitudes and behaviours (e.g., time use, consumer satisfaction, environmental pathogen contamination); health outcomes due to water‐related health infection (e.g., diarrhoeal infection, acute respiratory infections, gastro‐intestinal worm infections); other health outcomes, which are largely gendered (musculoskeletal disorder, reproductive tract infection, injury and psychosocial health); nutritional status, relating to water‐related disease and carer and children's time use; socioeconomic outcomes (e.g., education and cognitive development, income poverty); and mortality (Chirgin et al., submitted).

**Figure 2:**
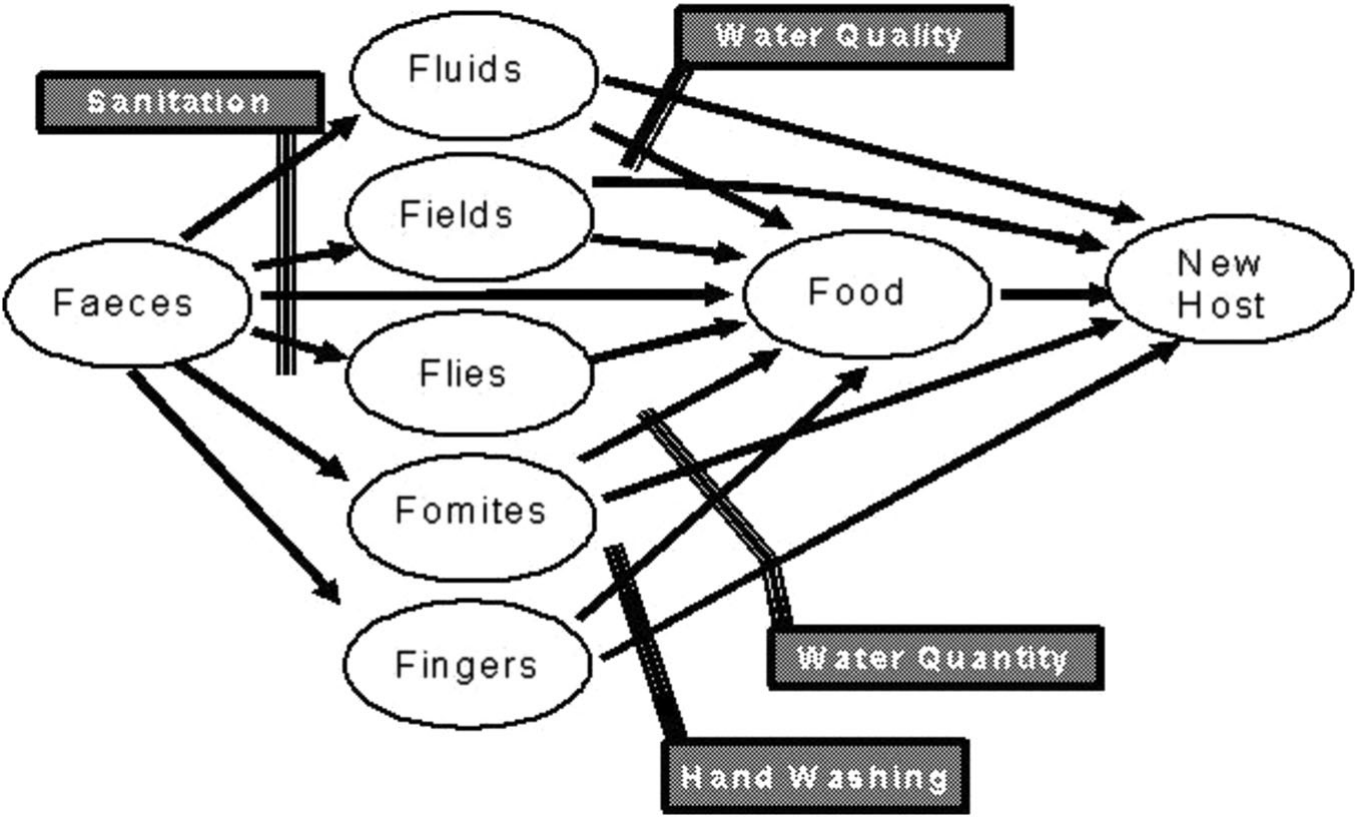
The “F”‐diagram showing faecal‐oral disease transmission. *Source*: Cairncross and Feachem (2018).

Figure 2 shows a theoretical depiction of the direct communication of faeco‐oral pathogens between individuals (Wagner & Lanoix, 1958). Later called the “F‐diagram” (e.g., Kawata, 1978), it shows the behavioural transmission routes for various water‐related diseases from faeces to future hosts via water (fluids), hands (fingers), arthropods (flies), soil (fields) and food. A sixth transmission route has since been identified, “fomites”—that is, objects acting as disease‐carrying vectors such as clothes, utensils, toys and furniture (Cairncross & Feachem, 2018). Implicit in the figure are three water‐related, faecal‐borne disease transmission routes: water‐borne diseases transmitted through ingesting infected water, water‐washed diseases transmitted through inadequate drinking water supply and hygiene (e.g., cholera, diarrhoeal disease, hepatitis, typhoid), and water‐based diseases transmitted by penetrating skin (e.g., schistosomiasis transmitted in water, and ascaris, hookworm and whipworm in contaminated soil) (White et al., 1972, p. 163).

The F‐diagram focuses on faecal‐borne diseases, but additional water‐related infections that are not faeces‐related. For example, water‐related insect vectors which pass on disease by breeding in water (e.g., chikungunya, dengue, malaria) (Cairncross & Feachem, 2018) are a major source of global mortality (Figure 1).

Figure 2 shows sanitation as a primary barrier to faecal‐related disease transmission, when excreta carrying faecal pathogens are eliminated from the environment or human consumption. Primary barriers also include hand washing and water quantity, important for stopping transmission primarily in the domestic domain (fingers and fomites). Due to faecal contamination of drinking water between source and point‐of‐use (POU), hygienic approaches may be needed to store clean water collected at source, or treat water for contaminants in the household (POU) (Fewtrell & Colford, 2004; Wright et al., 2004). Better access to water supply (quantity) may improve health by reducing contamination in the environment by enabling better personal hygiene (e.g., handwashing) and environmental hygiene (e.g., safe disposal of faeces). The secondary barrier is drinking water quality (Kawata, 1978). Factors such as environmental faecal contamination may prevent impacts from clean drinking water provision being realised due to the amount of time infants and children, who are the most susceptible to diarrhoeal disease, spend on the floor and putting their fingers in their mouths.^4^

**Figure 3:**
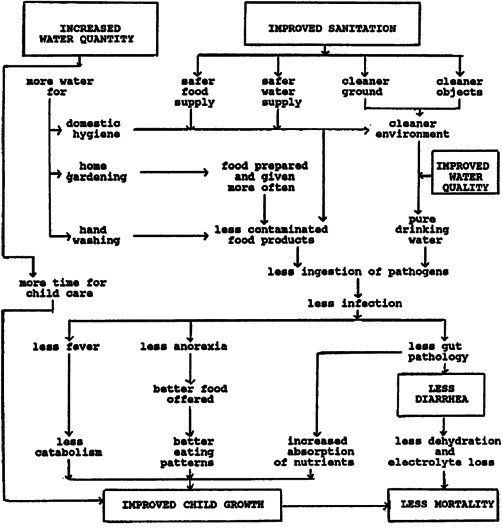
Relationship of improved water, sanitation and hygiene to diarrhoea, child growth and mortality among young children. *Source*: Esrey (1987).

Esrey (1987) presented a logic model showing the theoretical relationship between water supply, water treatment, sanitation and hygiene, on the one hand, and diarrhoeal disease, child nutritional status, and survival, on the other (Figure 3). That figure indicates that the routes from water supply and sanitation to survival operate through various intermediate quality of life outcomes relating to better hygiene practices (including hand‐ and food‐hygiene, and “fomites”) and child care, diarrhoeal disease and nutrition.

The figure is highly simplified and excludes underlying assumptions. Links in the causal pathway between interventions and outcomes are not automatic. For example, water treatments may not lead to less faecal contamination if the treatment technology itself is not efficacious in combating parasitic infections (Arnold & Colford, 2007). An example would be chlorination which is not effective against cryptosporidium, a common cause of diarrhoeal morbidity and mortality, especially among immunocompromised groups such as those living with HIV/AIDS (Havelaar et al., 2003; cited in Abubakar et al., 2007). And even an efficacious technology may not reduce contamination if used improperly, for example, where insufficient protective agents are applied to treat drinking water, or insufficient time available to purify water before ingestion. In the case of drinking water provided at source, there may be environmental contamination during transport (e.g., use of contaminated storage containers) or poor personal hygiene at POU (e.g., when contaminated hands are put in water storage containers) (Wright et al., 2004). Other factors limiting effectiveness are due to adoption, for example, users may dislike the odour and taste of chlorinated water.

Similarly, providing latrines may not necessarily lead to less open defaecation (Clasen et al., 2010), for various reasons such as the quality of facilities (cleanliness and smell) or concerns from pit owners about the frequency that the pit will need to be emptied. Nor may latrine provision lead to better health and nutrition if open defecation is still practised by some people in densely populated areas (Kar & Chambers, 2008). Latrines are not usually designed for or used by children, who may be afraid of going into dark places or of falling into the pit. This may be particularly problematic for reducing environmental contamination because children's excreta are more likely to contain infectious pathogens than adults' (Cairncross & Feachem, 2018), even though they may not be thought dangerous or offensive (Curtis et al., 1995; see also Majorin et al., 2019).

Preventive technologies tend to be adopted more slowly as benefits are difficult to observe (Rogers, 2005). This applies particularly to WASH technologies whose main benefit is to reduce diseases, the prevalence of which may typically be infrequent (or effects unobserved) outside of epidemics. For example, the incidence of diarrhoeal disease among study participants in low‐ and middle‐income countries (L&MICs) was around 10% in one systematic review (Waddington et al., 2009). An average reduction in risk of child diarrhoea by 30%, the typical pooled effect size found in meta‐analyses of WASH technology evaluations, would therefore only reduce the number of diarrhoeal days from 10% to 7% on average, if the measure were based on prevalence.^5^ Even a reduction in average risk by 50% for household water filtration, would reduce the typical child diarrhoeal risk from three episodes per year to 1.5 episodes (Clasen et al., 2015). In contrast, where the benefits of a technology are easily observed by those directly affected, such as poor women and children collecting water every day, and hence adoption likely to be rapid where it can be adequately provided, it is more likely that underinvestment in the technology would be explained by systemic undervaluation of the benefits and costs (including opportunity costs) for the affected groups, both by public authorities and household decision makers. Indeed, while health is the main preventive outcome for WASH, it is not a major motivating factor for WASH behaviours (Jenkins, 1999).

Sustaining impacts and achieving them at scale requires the continued wide acceptance and adoption of new technology, which may require additional promotional approaches. Sustainability and scalability of impacts are therefore central issues for policy and practice. Sustainability of impacts requires continued adherence by beneficiaries, solutions to “slippages” in behaviour and financial barriers to uptake, as well as technical solutions to ensure service delivery reliability. Scalability requires that impacts measured in small‐scale efficacy settings (the “ideal settings” measured in many field trials) are achievable in the context of programme effectiveness (“real world” settings) where fidelity of implementation becomes crucial (Bamberger et al., 2010). For example, hygiene information, education and behaviour change activities are usually a component of most, if not all, programme designs which aim to scale‐up service provision. However, there are concerns about whether these activities are being implemented in practice (Jimenez et al., 2014).

**Figure 4:**
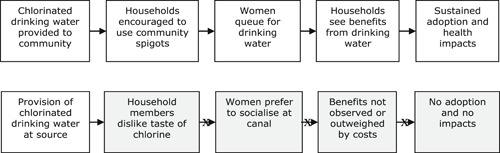
Programme theory and practice: public spigots in Egypt. *Source*: Author drawing on the description contained in Rogers (2005).

However, the effectiveness of WASH technology in preventing disease transmission depends on both the biological efficacy of the technology and its acceptability and use, or effectiveness, among consumers in the environment where it is based (Eisenstein et al., 2007). Acceptability and use in turn are determined by the WASH promotional intervention, which motivates behaviour change by triggering drives (e.g., disgust), emotions (e.g., status) or interests (e.g., curiosity) (Biran et al., 2014; Aunger & Curtis, 2016). Authors of diarrhoea efficacy studies have referred to lack of convenience and limited observability of health benefits in explaining why compliance rates may be low for household water treatment (Quick et al., 2002). As far back as the 1960s, Rogers (2005) documented the low level of use of public spigots in Egypt, despite government media campaigns warning people of the risks from drinking canal water. Qualitative research suggested various causes, including that users did not like the chemical taste of the chlorinated water, rumours that the chemicals were being used to control fertility, women preferring to gather water from the canal banks where they socialised, and long queues, and fighting in the queues, due to low water pressure (Figure 4).

### 1.4 Why this review is needed

#### 1.4.1 The policy debate and international targets

There is great interest in the impacts of WASH on child mortality in policy communities. This is in part due to the method of calculation of DALYs (Cairncross & Valdmanis, 2006), which sums years of life lost (YLL) and years lived with disability (YLD) associated with a particular exposure or disease. Every death attributed to infection, especially among children, is weighted heavily in YLL in the DALY calculation. In contrast, a calculation of YLD based on numbers of days experiencing diarrhoeal disease is rather smaller in endemic circumstances, since the typical child diarrhoeal risk among populations lacking access to clean drinking water may be three episodes per year (Clasen et al., 2015). For example, the recent GBD exercise estimates YLL for acute lower‐respiratory tract infections at over 1,300 deaths per 100,000 and diarrhoea at 960 deaths per 100,000 (GBD, 2016 Cause of Death Collaborators, 2017a). These are the third and fourth highest numbers of YLL to a single disease among all causes of mortality (and the highest among communicable diseases). In contrast, YLD were estimated at one‐tenth of the level of YLLs for diarrhoea (100 per 100,000) and around 1% (10 per 100,000) of YLLs for lower‐respiratory tract infections (GBD, 2017b).^6^

There has been broad consensus on the need for international targets to improve WASH technology access since the 1977 United Nations (UN) Water Conference at Mar del Plata and subsequent International Drinking Water Supply and Sanitation Decade of the 1980s (Jolly, 2004). The goal of that Decade, ratified by the Conference, was to provide adequate access to safe water and hygienic latrines to the population of the world by 1990 (Cairncross et al., 1980, p. xi). Yet, by 1990, only an estimated 76% of the global population were using an improved drinking water source and 54% used improved sanitation, as defined by the JMP (WHO/UNICEF, 2013). In 1990, the Convention on the Rights of the Child recognised the “right of the child to the enjoyment of the highest attainable standard of health… through the provision of… clean drinking water, taking into consideration the dangers and risks of environmental pollution” (Article 24, p. 57; cited in Jolly, 2004, p. 274).

The Millennium Declaration in 2000 included a water goal, and, following a declaration at the World Summit on Sustainable Development at Johannesburg in 2002, a sanitation goal was added (Jolly, 2004). The resulting Millennium Development Goal (MDG) 7 drinking water and sanitation targets were to halve (from 1990 levels) the proportion of people without sustainable access to safe drinking water and basic sanitation by 2015. The water indicator was later further defined as access to water from an improved source within 1 km of the household. This is roughly the time taken for a 30‐min round‐trip to collect water in the absence of queueing, which has been demonstrated as the time up to which basic needs for water supply can be reasonably met (Cairncross & Feachem, 2018; White et al., 1972). There are circumstances where it is likely that more than 30 min will be needed for 1 km roundtrips, such as mountainous or sandy terrain, or in water scarce regions where people may spend more time queuing at the water collection point than travelling to it (Dar & Khan, 2011).^7^ It is worth noting that the apparatus has been in place to monitor progress on water collection times at national (rural and urban) in most countries at least since the Demographic and Health Surveys (DHSs) Phase II (1988–1993) included a question on the time taken to “go there, fetch water, and come back” (Institute for Resource Development/Macro International, 1990). JMP has since defined improved drinking water as “basic” when it requires <30 min round‐trip to collect (Table 1).

The water target was declared met at the global level (WHO/UNICEF, 2013). However, by 2017, 144 million people used surface drinking water directly from a river, pond, canal or stream, 435 million people used unprotected wells, springs or other unimproved sources, and 206 million used improved water that required more than 30 min roundtrip to collect.^8^ There also remain big regional inequalities in access. In sub‐Saharan Africa, 416 million people use surface water, unimproved drinking water sources, or have limited access to improved services (requiring more than 30 min round‐trip to collect). In South Asia, 137 million use surface water, unimproved water or have limited services, and in East Asia, 165 million people use them. The biggest improvements in access to drinking water have been in Asia, but coverage for 2.14 billion people in East Asia and the Pacific and 1.65 billion in South Asia remains “basic.” This means improved drinking water is provided at the community level or, if provided on premises, the supply is unreliable or contaminated.

The target for the MDG sanitation indicator, defined as the use of unshared, improved sanitation, was missed at the global level and in most countries in South Asia and sub‐Saharan Africa by a wide margin (United Nations, 2015).^9^ Of the 1.4 billion people who defecate in the open or use unimproved sanitation, 505 million live in South Asia (of which 375 million are in India) and 546 million in sub‐Saharan Africa. A further 620 million share limited sanitation facilities with two or more households (233 million in South Asia, 188 million in sub‐Saharan Africa and 145 million in East Asia and the Pacific). By 2015, 4.5 billion people lacked access to safely managed sanitation, where excreta were disposed of safely in situ or offsite (UN Water, 2018).

The Agenda for Sustainable Development set new global targets for 2030, enshrined in the Sustainable Development Goals (SDGs).^10^ The SDGs include targets for both access to basic services, which is a necessary condition to improve quality of life outcomes, and use of improved drinking water and sanitation, which is the sufficient condition to improve them. The SDGs are more ambitious than the MDGs, aiming to “ensure the availability and sustainable management of water and sanitation for all” by 2030 (UN Water, 2018). This greater ambition is reflected in both the indicators being measured, going beyond “improved” to “safely managed” services (Table 1), and the targets, which in most cases require universality in coverage by 2030.^11^ This greater ambition may be necessary to achieve the population health and nutrition improvements long claimed (Cumming et al., 2019). The SDGs also incorporated targets for hand washing for the first time.

Reaching these targets will be challenging, and not just for sanitation and hygiene. For example, only 15 countries with <95% coverage are on track to achieve universal coverage of basic drinking water, only 14 countries with <95% coverage are on track for universal basic sanitation, and only 18 countries are on track to eliminate open defaecation (WHO/UNICEF, 2017). In 2016, the UN proclaimed 2018–2028 the International Decade for Action on Water for Sustainable Development.^12^ To provide universal coverage, including appropriately serving the most disadvantaged people, it will be necessary to promote effective interventions for different groups, particularly disadvantaged groups who are most likely to be hidden from coverage, in the contexts in which they are used in private and public realms (e.g., schools, health facilities, places of transit, work, commerce and recreation, streets and fields).

It may appear difficult to understand the continued limited access to and use of WASH in spite of these commitments, when the technologies and resources exist to provide everyone with safely managed WASH, and improved WASH provides the foundation for combating communicable diseases like diarrhoea which is endemic in low‐income communities, killing millions every year. Improved WASH is also important for blocking infectious disease transmission in epidemics, such as cholera outbreaks and the COVID‐19 pandemic (Howard et al., 2020).

At least part of the reason is due to competing priorities among decision makers, whether they are policymakers at the top, service providers, or service users at the bottom. In order to stand a chance of meeting universal SDG targets, decision makers need access to evidence on what are the most effective ways to provide access to and promote use of WASH services, in particular contexts, and for specific groups.

#### 1.4.2 Existing systematic evidence

There has been an explosion in the production of studies like randomised controlled trials (RCTs) that are able to attribute changes in diarrhoeal disease to WASH interventions (Chirgwin et al., submitted). Correspondingly, many systematic reviews and meta‐analyses have synthesised the effects of these studies in L&MICs. The earliest reviews covered faeces‐related infections associated with water and sanitation provision including diarrhoea (Esrey et al., 1985, 1991). Esrey concluded that “safe excreta disposal and proper use of water for personal and domestic hygiene appear to be more important than drinking water quality in achieving broad health impacts” (Esrey et al., 1991, p. 31).

Fewtrell and Colford (2004), Fewtrell et al. (2005) updated Esrey et al., (1985, 1991), concluding that both hygiene education and water quality interventions reduced the risk of diarrhoea disease by about 40% each in L&MICs, while sanitation provision or water supply reduced the risk by only around 20% each. A meta‐analysis conducted by Clasen et al. (2006, updated in 2015) also supported the finding that water treatment at POU, particularly filtration, was more effective in reducing diarrhoea risk than other types of water improvements. These findings were replicated in Hunter (2009) and the WHO (Wolf et al., 2014, 2018). Interventions appeared to be more effective when a safe water storage container was also provided (Clasen et al., 2015), as it is, for example, in filtration devices from which water is accessed through a tap. A few meta‐analyses of higher quality studies also found that piped water to households significantly reduced diarrhoea morbidity (Waddington et al., 2009; Wolf et al., 2018). Wolf et al. (2018) also defined piped water according to reliability and quality, finding big impacts, although only one study measured it.

The evidence on sanitation is mixed. First, until the last decade there were few impact evaluations of sanitation impact covering more than a small number of clusters. Second, previous reviews did not take clustering into account. Thus, earlier reviews estimated between 25% and 35% reductions in diarrhoea from sanitation (Fewtrell & Colford, 2004; Norman et al., 2010; Waddington et al., 2009; Wolf et al., 2018). Replacing on‐site sanitation with water‐based sewerage was estimated to reduce the incidence of diarrhoea by around 30%, though it may not always be a suitable solution given the maintenance costs (Norman et al., 2010). Meta‐analyses suggested hand‐hygiene interventions reduced reported diarrhoea morbidity by between 30 and 50% (Aiello et al., 2008; Cairncross et al., 2010; Curtis & Cairncross, 2003; Ejemot‐Nwadiaro et al., 2015; Waddington et al., 2009; Wolf et al., 2018). Soap provision appeared to be particularly effective (Aiello et al., 2009; Waddington et al., 2009).

A common finding from meta‐analysis of indirect study comparisons (that is, findings across different contexts) is that bundling WASH interventions together does not produce additional effects in comparison with single water, sanitation or hygiene interventions (Fewtrell & Colford, 2004). For example, the World Bank's Independent Evaluation Group (White and Gunnarsson, 2008, p. 17) concluded that “the health impact of combined methods has not been found to be stronger than any single approaches.”

However, there are concerns about the quality of evidence on the effectiveness of WASH interventions in reducing morbidity, due to concerns about self‐ and carer‐reported infection, particularly where survey participants are exposed to repeated measurement in open (nonblinded) trials (Schmidt & Cairncross, 2009; Zwane et al., 2011). One advantage of water treatment technology with respect to conducting trials is that it is possible to blind participants—for example, by providing the plastic bottle but no instructions about storage for ultraviolet (UV) filtration (Conroy et al., 1996). Schmidt and Cairncross (2009) reported that blinded studies of household water treatment estimated impacts that were not significantly different from zero. Other reviews of household water treatment trials have found smaller or null effects once double‐blinding was taken into account (Clasen et al., 2006, 2015; Hunter, 2009; Waddington et al., 2009; Wolf et al., 2018).

Others have noted that water treatment technologies were more effective where adherence was higher (Arnold & Colford, 2007; Clasen et al., 2015; Waddington et al., 2009). One review found that “water quality interventions conducted over longer periods tend to show smaller effectiveness, while compliance rates, and therefore impacts, appear to fall markedly over time” (Waddington et al., 2009; iii). Schmidt and Cairncross (2009) concluded that “widespread promotion of household water treatment is premature given the available evidence” (p. 986). There therefore has been, and still is, considerable controversy as to the role and scalability of water treatment interventions in combating diarrhoeal disease.

Issues affecting the quality of self‐reported diarrhoea morbidity may also affect hygiene interventions. Although no studies with double blinding of participants and outcome assessors have been conducted of hygiene interventions in L&MICs, blinding of outcome assessors is achievable, for example, where participants were provided children's reading material unrelated to hygiene (Luby et al., 2006). One systematic review found a smaller, but still statistically significant, 20% reduction in risk of diarrhoeal morbidity in blinded trials of hygiene (Ejemot‐Nwadiaro et al., 2015).

It appears to be increasingly common to adjust for lack of blinding using Bayesian meta‐analysis. Hunter (2009) proposed a bias correction procedure to water treatment studies drawing on coefficients from a meta‐epidemiology study, presented in Wood et al. (2008). In the updated Cochrane drinking water treatment review by Clasen et al. (2015), similar bias correction factors were also applied, although the authors noted that “we urge caution in relying on these adjusted estimates since the basis for the adjustment is from clinical (mainly drug) studies that may not be transferable to field studies of environmental interventions” (p. 9). Wolf et al. (2018) also adjusted household water treatment and hygiene interventions for bias due to lack of blinding, but not water supply and sanitation, arguing that water supply and sanitation have recognised benefits over and above health impacts, whereas water treatment and hygiene “usually aim exclusively to improve health which is apparent to the recipient” (p. 512). It is worth noting that the correction factor for hygiene studies is particularly large, yielding a highly imprecise estimate (odds ratio [OR] = 0.90, 95% confidence interval [CI] = 0.37, 2.17; 33 studies) that is much bigger than the bias from single blinding estimated by Ejemot‐Nwadiaro et al. (2015).

To summarise, a large number of systematic reviews and meta‐analyses of impact evaluations have linked WASH to diarrhoeal disease. The common outcome indicator collected in health impact evaluations and systematic reviews is diarrhoea morbidity. Morbidity is presumably collected as a proxy for diarrhoea mortality, since it is easier to measure for financial and ethnical reasons (Briscoe et al., 1985). However, it may be a poor proxy for diarrhoea mortality. Diarrhoeal disease prevalence—number of days with diarrhoea over a period—is thought to be more closely correlated with mortality than diarrhoea incidence—number of distinct diarrhoea spells over a period (Morris et al., 1996; Schmidt et al., 2011). In addition, morbidity estimates may be affected by censoring of data, particularly in observational studies and cluster‐RCTs where recruitment of individuals is done after randomisation, or in studies (including RCTs) where children of different ages, and therefore lengths of exposure, are followed‐up concurrently.

An alternative approach is to evaluate impacts on mortality. Meta‐epidemiological evidence suggests that bias due to self‐reporting is not problematic for all‐cause mortality, and to a lesser extent cause‐specific mortality (Savović et al., 2012, Wood et al., 2008), especially if taken from vital registration systems rather than reported by “verbal autopsy” in carer surveys. However, mortality measurement is complicated in prospective studies due to ethics and statistical power (Briscoe et al., 1985). It is unethical to let people die in the course of intervention research when oral rehydration salts (ORS) or medical treatment may be easily provided to severely ill children. Designing studies which can measure childhood mortality with statistical precision is also complicated as mortality is a sufficiently rare outcome outside of epidemics. It is possible to use the greater statistical power of meta‐analysis to pool findings from studies in order to estimate statistically precise effects of rare outcomes (Waddington et al., 2018), which is the approach taken in this systematic review.

## 2 OBJECTIVES

The objective of the systematic review is to answer two main review questions:

(1) What are the effects of improved water, sanitation and hygiene access in L&MICs on:
  - all‐cause mortality in childhood?
  - diarrhoea and infection‐related mortality in childhood?
(2) To what extent do the effects vary by contextual factors, such as geographic location and baseline environmental risk, factors relating to the study participants, such as age, sex and immunocompromised status and factors relating to the implementation of the study itself, including design, risk of bias assessment and length of follow‐up?

## 3 METHODS

### 3.1 Criteria for considering studies

Table 2 summarises the criteria for inclusion of populations, interventions, comparators, outcomes, study designs, settings, language and time frame, which are discussed in this section.^13^

**Table 2:** Summary of inclusion criteria

Criteria	Definition
Populations	Human populations in low‐ and middle‐income countries, as defined by the World Bank at the time the research was carried out. Populations of any age, sex, gender, disability or socio‐economic status were included. Populations in epidemics (e.g., cholera outbreak) were excluded
Interventions	Studies of WASH interventions and technologies (exposures) were eligible. Interventions included demand side (behaviour change communication, subsidies, microloans, legal measures), supply side (direct hardware provision, privatisation and nationalisation, small‐scale independent provider involvement, improved operator performance), or combinations of demand and/or supply side (decentralisation). Eligible WASH technologies included water supply, water treatment and safe storage, sanitation, and/or hygiene
Comparators	Impact evaluations where the comparison/control group received no intervention (standard WASH access), a different WASH intervention, a double‐blind placebo (e.g., nonfunctioning water filter), a single‐blind (e.g., school textbooks), or a pipeline (wait‐list)
Outcomes	Mortality in childhood: all‐cause and cause‐specific mortality due to diarrhoea and infection. Mortality may be measured as a primary study outcome, or harvested from prospective trial participant flow diagrams
Study design	Randomised controlled trials, prospective and retrospective nonrandomised studies, natural experiments and observational studies with control for confounding (e.g., case‐controls)
Settings	WASH provided for use in the household, community, school, or health care facility
Language	Studies in English, French, Spanish and Portuguese. Studies in other languages were included where an English translation was available
Time frame	Studies published at any time; no study was excluded based on date of publication

#### 3.1.1 Types of studies

Eligible studies are impact evaluations, defined as programme evaluations or field experiments that used quantitative approaches applied to experimental or observational data to measure the average effect of participating in a WASH programme relative to a control or comparison group (counterfactual) representing what would have happened to the same group in the absence of the programme. Eligible impact evaluations may also test different intervention mechanisms or technologies (i.e., active controls).

The following study designs will be included:

(1) Prospective quantitative evaluations where participants were assigned to intervention(s) at individual or cluster levels:
  a. RCTs with randomised assignment of units at individual and household level (e.g., Han & Hlaing, 1989), or with cluster assignment at a higher level (village, township, school or health facility) (e.g., Clasen et al., 2015; Pickering et al., 2015), quasi‐RCTs using quasi‐randomised assignment of units (e.g., alternation of clusters listed alphabetically), and studies using randomised encouragement, providing promotional information about an intervention or technology that is universally available (e.g., Devoto et al., 2012).
  b. Nonrandomised studies (NRSs) with assignment of units based on practitioner or participant selection and contemporaneous measurement of outcomes by investigators at pre‐ and posttest in treatment and comparison groups,^14^ or contemporaneous measurement by investigators in treatment and comparison group at posttest only. These include prospective cohort studies (e.g., Ryder et al., 1985), studies using methods such as statistical matching (e.g., propensity score matching [PSM]) (e.g., Reese et al., 2019), or direct control for confounding in adjusted analysis (e.g., Cole et al., 2012). Cross‐sectional studies that analysed the relationship between WASH technology interventions and outcomes, which compared self‐selected participants within the same group, but did not use any methods to control for confounding (e.g., Gross et al., 1989) were excluded.
  c. NRSs with measurement by investigators in treatment group at least six time points pre‐ and posttest (interrupted time‐series) (Fretheim et al., 2015).
  d. Cross‐over trials where treatment and control or comparison are swapped (e.g., Kirchhoff et al., 1985).
(2) NRSs designed retrospectively—that is, after intervention had occurred—with selection on observables, including nonrandomised pipeline design, studies using cross‐section data, and studies using panel data or pseudo‐panels of repeated cross‐sections with an intervention and comparison group, using methods to match individuals and groups statistically or control for observable confounding in adjusted analysis (e.g., Galiani et al., 2005).
(3) Case‐control designs, and other types of studies of WASH exposures, will be included (e.g., Hoque et al., 1999; Victora et al., 1988).
(4) Natural experiments designed retrospectively with selection on unobservables:
  a. Natural experiments using exogenous treatment assignment rules, including randomised natural experiments (with assignment by public lottery), and natural experiments where assignment was by random errors in implementation (e.g., Morris et al., 2004).
  b. Regression discontinuity designs (RDDs) with prospective assignment to intervention and comparison groups based on a threshold on a continuous variable (e.g., number of cases of disease in a community, poverty index) (e.g., Spears, 2013; Ziegelhöfer, 2012) or a physical threshold such as an administrative boundary (Villar & Waddington, 2019).
  c. Studies using multistage or multivariate approaches with identification of compliers based on exogenous variation (e.g., instrumental variables) or double‐differences (e.g., Geruso & Spears, 2018).

Study designs without a comparator receiving a different intervention or service (e.g., Israel, 2007) will be excluded, as will studies that did not control for confounding (e.g., Wagner & Lanoix, 1959; World Bank, 1998). Studies, or components of studies, that collected and analysed purely qualitative evidence will also be excluded.

#### 3.1.2 Types of participants

Eligible participants are children aged under 20 in a L&MIC, as defined by the World Bank at the time the research was carried out. This includes children of any age, sex, gender, disability, immunocompromised state, or socioeconomic status, provided the study was conducted in endemic conditions found regularly in L&MICs. Hence, studies that were conducted under outbreak conditions, such as cholera epidemics, will be excluded (e.g., Daniels et al., 1999; Snow, 1855).

#### 3.1.3 Types of interventions

Studies will be included that measure receipt of a clearly defined WASH intervention, or use of a WASH technology for household and personal consumption. Interventions will be excluded in food hygiene in the workplace such as a market (e.g., Sobel et al., 1998), methods to control faecal contamination by animals in the yard (e.g., Oberhelman et al., 2006), and vector control methods such as fly spraying (e.g., Chavasse et al., 1999; Emerson et al., 1999). Interventions primarily supporting farms or businesses such as dam construction (e.g., Duflo & Pande, 2007) will also be excluded, as will interventions for groundwater or irrigation management (e.g., Meenakshi et al., 2013). Likewise, flood and drought management interventions and river, lake, coastal zone and wetlands management will be omitted. Finally, cointerventions with a major non‐WASH component will be excluded, such as those providing deworming chemotherapy (e.g., Miguel & Kremer, 2004) and nutrition interventions (e.g., Humphrey et al., 2019), although any WASH‐only trial arms without co‐interventions of such studies are eligible (e.g., Luby et al., 2018; Null et al., 2018).

#### 3.1.4 Types of settings

WASH provided for use in the household, community, school, or health care facility. Studies on medicalised hygiene (such as sterilising wounds) will be excluded.

#### 3.1.5 Types of outcome measures

The two primary outcomes are all‐cause mortality and mortality due to infection including diarrhoeal disease, usually defined as three or more water stools in a 24‐h period (Bacqui et al., 1991). All‐cause mortality may be defined by carers in self‐report, and/or clinicians. Mortality due to diarrhoea and other infections may be defined by carers in verbal autopsy and/or clinicians, or collected from vital registries.

Outcomes data will be taken from two sources. The first is in studies that report childhood mortality as a primary outcome like case‐control studies and those using DHS data (e.g., Charmarbagwala et al., 2004). However, as noted above, for prospective studies, mortality measurement is complicated ethics and statistical power (Briscoe et al., 1985). Mortality data are recoverable from prospective studies that report losses to follow‐up (attrition) in sample populations due to mortality, per CONSORT standards (Moher, 1998; Moher et al., 2010). These studies will therefore form the second source of evidence on childhood mortality.

Eligible outcomes relate to a WASH intervention mechanism or exposure. For example, where some programme evaluations of CDD—an approach that is used to provide projects in multiple sectors such as infrastructure, education and health—do not give estimates of outcomes separately for WASH projects, these outcomes will be excluded.

#### 3.1.6 Publication language and date

In addition to English, studies published in French (Messou et al., 1997), Spanish (Instituto Apoyo, 2000) and Portuguese (e.g., Rasella, 2003) will be included. Studies published in other languages are eligible if an English translation is available. Studies published at any time are eligible, hence no study will be excluded based on date of publication.

### 3.2 Search methods for identification of studies

The review is being done based on an evidence and gap map of WASH programmes in L&MICs (Chirgwin et al., submitted), for which searches and coding of outcomes were done originally in 2018, and updates performed in 2020.

#### 3.2.1 Electronic searches

Electronic searches include the following academic databases: CAB Abstracts, CAB Global Health, Cochrane Library, Econlit, Embase, ERIC, Ovid MEDLINE, Popline, Proquest Social Sciences Collection. In addition, searches include completed trials identified in the following trial registries (OpenTrials, WHO International Clinical Trials Registry Platform, 3ie's RIDIE, and AEA RCT registry). An example search string is given in Appendix A.

#### 3.2.2 Searching other resources

Organisational website and repository hand searches include: the Impact Evaluation Repository of the International Initiative for Impact Evaluation (3ie), the Asian Development Bank, African Development Bank, Inter‐American Development Bank, J‐PAL evaluations, Innovations for Poverty Action, CEGA water and sanitation research projects, DFID Research for Development, IMPROVE International, IRC (WASH),^15^ Oxfam, UNICEF, US Agency for International Development, WaterAid, and the World Bank Development Impact Evaluation (DIME) and IEG. Finally, the bibliographies of all included systematic reviews were checked to identify additional primary studies and systematic reviews.

Reference lists of books, reports and meta‐evaluations to capture studies missed in electronic searches, particularly early studies, include: White et al. (1972), Saunders and Warford (1976), Feachem et al. (1978), Cairncross et al. (1980), WHO (1983), Khan et al. (1986), Briscoe et al. (1986), Charmarbagwala et al. (2004), White and Gunnarsson (2008) and Esteves Mills and Cumming (2016).

### 3.3 Data collection and analysis

#### 3.3.1 Selection of studies

Studies identified for selection are based on searches which were done at title and abstract by two authors working independently, using EPPI‐reviewer's machine learning software (Thomas et al., 2010), as presented in Chirgwin et al. (submitted). Selection of studies at full text was done by two authors working independently (Chirgwin et al., submitted).

#### 3.3.2 Data extraction and management

A standardised data extraction form will be used to collect descriptive data from all the included studies. This includes country, location (rural, urban, nationwide), participant age‐group, WASH intervention and technology, study design, environmental contamination as represented by community water and sanitation access at baseline, risk of bias, effect size and standard error.

#### 3.3.3 Assessment of risk of bias in included studies

Comprehensive critical appraisal will be done, including risk of bias and publication bias assessments (Dickersin, 1990). Study methods will be critically appraised using a risk of bias tool developed for this review (Appendix B), drawing on existing approaches (Eldridge et al., 2016; Higgins et al., 2016; Hombrados & Waddington, 2012; Sterne et al., 2016; Waddington et al., 2017; Jimenez et al., 2018). The following categories of bias will be assessed:

1. *Confounding*: baseline characteristics are similar in magnitude, unbalanced characteristics are controlled in adjusted analysis; for randomised approaches, adjustments to the randomisation were taken into account in the analysis (e.g., stratum fixed effects, pairwise matching variables); time‐varying confounding such as differential adherence in sustained interventions.
2. *Selection bias into the study*: randomisation approach and allocation concealment for individual and cluster‐randomisation. For NRSs, timing of follow‐up.
3. *Attrition or selection bias out of study*: total attrition and differential attrition across study groups (presentation of average characteristics across treatments and comparisons, and reasons for losses to follow‐up). In cluster designed studies, where respondents are not followed over time, assessment is needed of the sampling strategy.
4. *Departures from intended intervention due to performance bias*: no‐shows and cross‐overs, addressed using intention‐to‐treat (ITT) or the complier average causal effect; spillover effects addressed through geographical distance between treatment and control or comparisons; differential contamination by external programmes (treatment confounding) addressed through information about adherence behaviour.
5. *Departures from intended intervention due to motivation bias*: observational data versus experimental data with clear link to intervention (informed consent); repeated measurement (frequency and regularity of survey rounds); Hawthorne, John Henry effects and survey effects (Zwane et al., 2011).
6. *Errors in measurement of intervention and outcome*: length of recall, definition of intervention and outcome, timing of data collection (seasonality, or seasonal variation accounted for some other way), method of data collection (observed versus reported), blinding of outcome assessors and, where possible, participants.
7. *Biases in analysis and reporting*: pre‐analysis plan or study protocol, reporting ITT alongside other estimators, blinding of data analysts.
8. *Unit of analysis error*: methods used to adjust standard errors to account for correlation of observations within clusters (e.g., cluster‐robust standard errors).

It is important to recognise that risk of bias will refer to the likelihood of bias in the estimated mortality rate (MR), which may be collected from study participant flow, as opposed to the overall risk of bias in the study for the other (primary) outcomes of interest. MRs will be computed over a standard period, as mortality measurements will increase over longer exposure periods, all else equal.^16^ For example, Gebre et al. (2011) and Siegel et al. (2004) used the following calculation for ${\text{CMR}}_{j}$, the crude MR in study *j* per 1,000 person‐years at risk:

(1) $${\text{CMR}}_{j}=\frac{D_{j}}{\frac{t_{j}}{12}\left(N_{j}-\frac{D_{j}+M_{j}}{2}\right)}\times 1000,$$

where *D*_*j*_ is the number of deaths, *t*_*j*_ is the study follow‐up period in months, *N*_*j*_ is the baseline sample size, and *M*_*j*_ is the number of people who permanently migrated out of the study area over the follow‐up period. This will be applied to data collected from included studies. Age‐specific MRs for children may also be calculated by replacing Equation (1) with the numbers of deaths and population shares among the specific age groups. Cause‐specific MRs will be calculated by replacing *D*_*j*_ with numbers of deaths attributed to diarrhoea and/or infectious diseases, determined by recalled verbal autopsy or taken from vital registration data. An important issue affecting crude death rate calculations is that they are right‐censored; that is, where data are collected contemporaneously among participants regardless of age, children born into the study and younger children have completed shorter durations than older children (e.g., White et al., 2005). This causes downwards bias in the estimate of mortality in any single trial arm, although the bias may be less problematic in randomised trials with contemporaneous data collection across arms. In these cases, the age‐specific MR per 1000 live births may be calculated, which is not susceptible to censoring:

(2) $${\text{MR}}_{j}=\frac{D_{j}}{\left(B_{j}-B_{j}^{D}\right)}\times 1000,$$

where $B_{j}$ is the number of live births and $B_{j}^{D}$ the number of still‐births.

#### 3.3.4 Measures of treatment effect

The main estimate of treatment used in this review will be the OR. OR is calculated from the two‐by‐two frequency table:

(3) $$\text{OR}=\frac{p_{t}/(1-p_{t})}{p_{c}/(1-p_{c})},$$

where *p*_*t*_ is the proportion in the treatment group and *p*_*c*_ the proportion in the comparison group. Where studies use regression methods, OR will be calculated as:

(4) $$\text{OR}=\frac{{(y}_{c}+b)/(1-{(y}_{c}+b))}{y_{c}/(1-y_{c})},$$

which makes use of $p_{t}=y_{c}+b$, where $y_{c}$ is the outcome mean in the control and *b* the regression coefficient on the treatment variable. In such circumstances, the standard error of the logarithm of OR is given by:

(5) $$\mathrm{se}\left(\ln\text{OR}\right)=\sqrt{\frac{1}{{n_{t}(y}_{c}+b)}+\frac{1}{{n_{t}(1-y}_{c}-b)}+\frac{1}{{n_{c}y}_{c}}+\frac{1}{n_{c}(1-y_{c})}}.$$

Some studies report the risk ratio, RR:

(6) $$\text{RR}=\frac{p_{t}n_{t}/n_{t}}{p_{c}n_{c}/n_{c}}=\frac{p_{t}}{p_{c}},$$

with standard error of the natural logarithm of RR given by:

(7) $$\mathrm{se}\left(\ln\text{RR}\right)=\sqrt{\frac{1}{n_{t}p_{t}}-\frac{1}{n_{t}}+\frac{1}{n_{c}p_{c}}-\frac{1}{n_{c}}},$$

where treatment and control risks are available, RR will be transformed into OR using:

(8) $$\text{OR}=\text{RR}\frac{1-p_{c}}{1-p_{t}},$$

where risks are not given, assumed risks, $\hat{p_{t}}$ and $\hat{p_{c}}$, equal to the median treatment and control risks from any studies in the same country measuring that outcome, will be used^17^:

(9) $$\text{OR}=\text{RR}\frac{1-\hat{p_{c}}}{1-\hat{p_{c}}\text{RR}},$$

where the hazards ratio is used, it will be converted into RR using the following transformation (Shor et al., 2017):

(10) $$\text{RR}=\frac{1-e^{\text{HR}\ln(1-p_{c})}}{p_{c}}.$$

Inserting Equation (10) into (9), it can be shown that:

(11) $$\text{OR}=\frac{1-p_{c}+(p_{c}-1)e^{\text{HR}\ln(1-p_{c})}}{-p_{c}e^{\text{HR}\ln(1-p_{c})}},$$

where 95% CIs are reported instead of *t* or se(b), the following will be used to calculate the standard error (Higgins & Green, 2011):

(12) $$\mathrm{se}(\text{OR})=e^{\frac{{\ln(\text{CI}}^{U})-\ln({\text{CI}}^{L})}{3.92}},$$

where ${\text{CI}}^{L}$ and ${\text{CI}}^{U}$ are, respectively, the lower and upper limits of the 95% CI.

Where studies report independent treatment and control arms, data for mortality from each treatment‐control comparison will be included. Where studies report multiple correlated effect sizes, for example, factorial studies comparing multiple treatment groups against a single control arm (e.g., Luby et al., 2018; Null et al., 2018), the control arms will be split by assuming the populations and deaths were evenly distributed between comparisons (affecting the precision of estimate, but not the effect size). This is to prevent studies with multiple results receiving greater weight than studies with only one effect estimate, or the inclusion of positively correlated effect sizes, which lead to underestimation of the summary variance (Borenstein et al., 2009a).

Where it is not possible to split control groups for multiple study arms, effect estimates may be combined into “synthetic effects,” by calculating an average effect, weighted by sample size, of the relevant pair‐wise comparisons in these studies, and variance accounting for the correlation between correlated comparison groups from the same study. The formula for the pooled variance is given as (Borenstein et al., 2009b):

(13) $$\text{Var}\left(\frac{1}{N}\sum_{i=1}^{N}{\ln(\text{OR})}_{i}\right)={\left(\frac{1}{N}\right)}^{2}\text{Var}\left(\sum_{i=1}^{N}{\ln(\text{OR})}_{i}\right)={\left(\frac{1}{N}\right)}^{2}\left(\sum_{i=1}^{N}{\mathrm{se}}_{i}^{2}+\sum_{i\neq j}^{N}r_{\mathrm{ij}}\sqrt{{\mathrm{se}}_{i}^{2}{\mathrm{se}}_{j}^{2}}\right),$$

where *N* is the total number of effects, and *r*_*ij*_ is the correlation between effects, calculated as the mean of the correlation of treatment groups and the correlation of the control groups, and *se*_*i*_ the standard errors. The correlation between control arms is assumed equal to 1 where the same control group was used as comparator and 0 otherwise. The correlation between treatment arms is assumed to be 0 when combining results from different treatment groups and 1 when combining results from the same treatment groups over time. When combining results across different individuals in the same treatment group the correlation is assumed 0.5, which estimates variance at the mid‐point between the two extreme cases of treating comparisons as independent (with correlation coefficient equal to 0) and most likely underestimating the variance, or treating them as perfectly correlated (correlation coefficient of 1) and most likely overestimating the variance (Waddington et al., 2009).

#### 3.3.5 Unit of analysis issues

Where study participants are grouped into correlated clusters of observations, the following error correction formula will be used to adjust standard errors (Higgins & Green, 2011; Waddington et al., 2012):

(14) $${\mathrm{se}(\ln(\text{OR}))}^{\prime }=\mathrm{se}(\ln(\text{OR}))\sqrt{1+\left(m-1\right)\rho },$$

where *m* is the average number of observations per cluster and ρ is the intra‐cluster correlation coefficient and 1+(m−1)ρ is the design effect (*Deff*). This adjustment will not be applied in clustered studies where outcomes of interest were defined at the cluster level (e.g., municipality MR).

#### 3.3.6 Dealing with missing data

Where deaths are not reported in any intervention arm, 0.5 will be added to all frequencies in order to calculate OR (Sanchez‐Meca et al., 2003).

Usually, the intra‐cluster correlation coefficient, ρ, is not reported. It will need to be imputed for studies not presenting cluster‐adjusted standard errors, or, for example, where effect sizes are calculated from participant flow diagrams. In studies that calculate test statistics using cluster‐robust standard errors, it may be possible to estimate the standard error using:

(15) $$\mathrm{se}(\ln\left(\text{OR}\right))\prime =\frac{\ln(\text{OR})}{t^{\prime }},$$

where *t*' is the test statistic for the effect size estimate (OR), calculated using cluster‐robust methods. Where the study does not use cluster‐robust methods, the value of ρ may be imputed using the following approach. The variance of *OR*, is calculated as:

(16) $$V(\text{OR})={\mathrm{se}\left(\text{OR}\right)}^{2}.$$

Inserting Equation (16) into (14) and rearranging gives^18^:

(17) $$\rho =\left(1-\frac{V\left(\text{OR}\right)\prime }{V\left(\text{OR}\right)}\right)\frac{1}{m-1},$$

where *V*(OR)' is calculated as the square of Equation (15) and *V*(OR) the square of Equation (5).

#### 3.3.7 Assessment of heterogeneity

A measure of relative heterogeneity—the proportion of variance due to variation in the “true” effects over sampling variation, or *I* ^2^ (Higgins & Thompson, 2002)—will be calculated (Borenstein et al., 2017):

(18) $$I^{2}=\frac{\tau^{2}/k}{s_{\mathrm{RE}}^{2}}=\frac{\tau^{2}}{\frac{\sigma^{2}}{n}+\tau^{2}},$$

where $\tau^{2}$ is the estimated between‐study variance, *k* is the number of studies, and $s_{\mathrm{RE}}^{2}$ is the random effects average variance; under the assumption of equal study variance and sample size, this is equal to the within‐study variance $\frac{\sigma^{2}}{n}$ plus the estimated between‐study variance variance $\tau^{2}$ (Borenstein et al., 2009a). *I* ^2^ is usually expressed as a percentage rather than a proportion. Absolute heterogeneity will be measured as the between‐study variance using the method of DerSimonian and Laird (1986) (Borenstein et al., 2017):

(19) $$\tau^{2}=\max\left\{0,\frac{Q-\mathrm{df}}{\sum_{i=1}^{k}w_{i}-\frac{\sum_{i=1}^{k}w_{i}^{2}}{\sum_{i=1}^{k}w_{i}}}\right\},\text{where}\;Q=\sum_{i=1}^{k}w_{i}{\left({\hat{b}}_{i}-\hat{\beta }\right)}^{2}\sim \chi_{\mathrm{df}=k-1}^{2},$$

where $\tau^{2}$ is artificially constrained at zero if the value falls below zero (since a variance cannot be <0), and *Q* is the inverse‐variance weighted sum of squares of the difference between effect sizes ${\hat{b}}_{i}$ and their estimated mean $\hat{\beta }$. *Q* is a statistic that follows the *χ* ^2^ distribution with degrees of freedom df=k−1, where *Q* represents the observed variation and df the expected variation based on sampling error alone.

#### 3.3.8 Assessment of reporting biases

Publication bias will be assessed using two methods. Direct tests for publication bias will be done in meta‐regression accounting for whether the study was published in a peer‐review journal, or another publication route such as a working paper or organisational report. Indirect testing of small study effects will use inspection of contour‐enhanced funnel graphs (Peters et al., 2008) and formal regression tests (Egger et al., 1997). These tests are based on the assumption that there are weaker incentives for researchers and journals to publish smaller sample studies that do not show significant findings, because the cost of such studies is less and/or that authors of underpowered (small‐sample) studies are more likely to undertake exploratory analysis (called “p‐hacking”) in order to obtain publishable results.

#### 3.3.9 Data synthesis

The sample requirements to estimate effects on mortality with statistical precision would usually be beyond what is possible in these studies. The approach taken in this systematic review will therefore be to take advantage of the greater power afforded by statistical meta‐analysis, to attempt to estimate precise pooled effects. Inverse variance weighted random effects meta‐analysis will be used to synthesise the findings. A standard approach to meta‐analysis will be followed, including sensitivity analysis by risk of bias, subgroup analysis by mortality causation.

The random effects pooled effect is calculated as the expected mean effect across the distribution of population effects, using a modified weighted average of the inverse of the variance incorporating two sources of sampling error—within‐study and between‐study variation. Each study weight is equal to the inverse of the within‐study error variance of the individual study $s_{i}^{2}/n_{i}$ plus the estimated between‐study variance $\tau^{2}$. Since the weight for a single study is equal to the inverse of the sum of the within and between study variances, the expected variance of the random effects average $s_{\text{RE}}^{2}$ is the inverse of the sum of the weights across the studies (Borenstein et al., 2009a):

(20) $$s_{\text{RE}}^{2}=\frac{1}{\sum_{i}^{k}\frac{1}{s_{i}^{2}/n_{i}+\tau^{2}}}$$

#### 3.3.10 Subgroup analysis and investigation of heterogeneity

Subgroups and moderator variables will be collected based on what is theoretically associated with mortality. Subgroups may include children of different age (Butz et al., 1984), sex and socioeconomic status, and mortality causation (all‐cause versus diarrhoea or other infectious diseases). Moderators will include WASH intervention technology, environmental risk as determined by water and sanitation availability to capture community threshold effects (Shuval et al., 1981), location, study design, risk of bias and length of follow‐up (Waddington et al., 2009). Baseline water and sanitation will be determined by the type that was most frequently used in the control or comparison group. Following Fewtrell and Colford (2004), where the study does not report the baseline assessment, the value will be imputed for the relevant country, location and year from the Joint Monitoring Programme dataset.

Both bivariate moderator analysis and multivariate meta‐regression will be used to investigate heterogeneity. For meta‐regression, a general‐to‐specific approach will be used to determine the optimum meta‐regression specification (Mukherjee et al., 1997).

#### 3.3.11 Sensitivity analysis

Sensitivity of the findings will be assessed for outliers and by stratifying meta‐analysis for those studies which included mortality as a primary outcome, versus those reporting mortality in participant flow.

## ROLES AND RESPONSIBILITIES

- Content: Hugh Waddington and Sandy Cairncross are responsible for content.
- Systematic review methods: Hugh Waddington and Sandy Cairncross are responsible for systematic review methods.
- Statistical analysis: Hugh Waddington is responsible for statistical analysis.
- Information retrieval: Hugh Waddington and Sandy Cairncross are responsible for information retrieval, based on searches designed by John Eyers, Information Retrieval Specialist with Campbell IDCG.

## SOURCES OF SUPPORT

The review is not supported financially, but is based on searches for an evidence and gap map that was funded by the Water Supply and Sanitation Collaborative Council (WSSCC).

## DECLARATIONS OF INTEREST

The authors have no vested interest in the outcomes of this review, nor any incentive to represent findings in a biased manner. Sandy Cairncross has been involved in the development of sanitation and hygiene interventions, Sandy Cairncross has also contributed to included impact evaluations, and Sandy Cairncross and Hugh Waddington have led previous systematic reviews and meta‐analyses on WASH and diarrhoea.

## PLANS FOR UPDATING THE REVIEW

Hugh Waddington will be responsible for updating the review, which will be done once financial support is available.

## Footnotes

^1^
Hygiene and water supply are also likely to be key blocks to the transmission of coronavirus disease 2019 (COVID‐19), a type of acute respiratory tract infection (Howard et al., 2020).

^2^
A fourth component, not discussed here, is the suitability of the intervention for particular groups including disadvantaged people (e.g., children, pregnant women, elderly and disabled people).

^3^
There are also intermediate steps on the sanitation ladder not listed in Table 1; for example, where there is no fixed place of sanitation but some attempt to remove faeces from exposure to others such as “cat sanitation” (Waterkeyn and Cairncross, 2005).

^4^
The F‐diagram relates to faecal‐borne pathogen related disease transmission. Noninfectious waterborne diseases, such as arsenicosis and fluorosis, caused through chemical contamination of water, are increasingly recognised as a source of human morbidity and mortality (Dar & Khan, 2011).

^5^
Diarrhoeal disease is usually measured as the risk, incidence, or prevalence. Risk measures the probability of being ill during the measurement period. Incidence density or rate measures the average risk over the measurement period measured in average number of discrete disease spells. Longitudinal prevalence is more closely associated with duration of illness, usually measured as the proportion of days of illness during the measurement period. Longitudinal prevalence of diarrhoea is preferred on theoretical grounds and empirically is more strongly associated with child mortality and weight gain than incidence (Morris et al., 1996). Different technologies may also affect measures of incidence and prevalence differently. For example, Gross et al. (1989) noted that hygienic practices such as removal of faeces from the yard may have greater impact on spell duration than incidence.

^6^
In addition, road injuries caused the fifth biggest numbers of YLL at 817 per 100,000 (of which pedestrian road injuries contributed 290 per 100,000) and were in the top 20 causes of YLD at around 200 per 100,000 (pedestrian injuries contributing one‐quarter of these). While musculoskeletal disorders caused 31 YLL per 100,000, lower back and neck pain was the biggest single cause of YLD (over 1,000 per 100,000) and other musculoskeletal disorders were the seventh highest (over 500 YLD per 100,000). Animal contact was estimated to contribute 58 per 100,000 YLL and around 30 YLD per 100,000.

^7^
A second issue with the water target, noted by Dar and Khan (2011), occurs where drinking water contaminated by chemicals may cause noninfectious diseases like arsenicosis or fluorosis.

^8^
WASH access and use data from https://washdata.org/

^9^
This relatively “uneven progress” in reaching WASH sector targets was in part due to the sanitation indicator, defined as unshared by households, being harder to reach than the water indicator, which included shared facilities at the community level (Cumming et al., 2014).

^10^
See http://www.un.org/sustainabledevelopment/sustainable-development-goals/

^11^
Unlike other targets which specify 2030, the target for ODF was originally specified for 2025 (Hutton & Varughese, 2016).

^12^

https://www.unwater.org/new-decade-water/ (accessed November 17, 2020).

^13^
This protocol has also been registered with Prospero: “Water, sanitation and hygiene (WASH) for reducing mortality in childhood in low‐ and middle‐income countries,” CRD42020210694. Available at: https://www.crd.york.ac.uk/prospero/display_record.php?RecordID=210694 (accessed December 3, 2020).

^14^
This designation also applies to RCTs with noncompliance that are analysed using ATET.

^15^
IRC was originally known as the International Reference Centre for Community Water Supply until the mid‐1980s when it changed its name to IRC International Water and Sanitation Centre, but as of 2014 simply goes by IRC. We use IRC (WASH) to distinguish it from the International Rescue Committee.

^16^
This is particularly important for comparative measures of MRs (effect sizes) that are time sensitive, such as risk differences, but less important for ratio estimates. However, follow‐up length will be collected from studies and included in meta‐regression analysis as it has been shown to be correlated with effect sizes in a previous meta‐analysis of diarrhoea morbidity (Waddington et al., 2009).

^17^
The formula to transform RR into OR used by Clasen et al. (2015), taken from Higgins et al. (2011), is: $\text{RR}=\frac{\text{OR}}{1-\hat{p_{c}}+\hat{p_{c}}\text{OR}}$, where $\hat{p_{c}}$ represents the estimated control risk.

^18^
Schmidt et al. (2011) presented another way to calculate $\text{Deff}=\frac{V\left(\text{OR}\prime \right)}{V\left(\text{OR}\right)}.$ However, this method does not allow adjustment by studies' known numbers of clusters and observations within clusters. Hence, where *m* is known, Equation (17) will be the preferred means of calculating ρ.

## APPENDIX A EXAMPLE SEARCH STRING

The example search string is formatted for Ovid MEDLINE(R).

1. Developing Countries.sh,kf. (79987)
2. Africa/or Asia/or Caribbean/or West Indies/or South America/or Latin America/or Central America/(69677)
3. (Africa or Asia or Caribbean or West Indies or South America or Latin America or Central America).tw. (151966)
4. (Afghanistan or Albania or Algeria or Angola or Argentina or Armenia or Armenian or Azerbaijan or Bangladesh or Benin or Byelarus or Byelorussian or Belarus or Belorussian or Belorussia or Belize or Bhutan or Bolivia or Bosnia or Herzegovina or Hercegovina or Botswana or Brazil or Bulgaria or Burkina Faso or Burkina Fasso or Upper Volta or Burundi or Urundi or Cambodia or Khmer Republic or Kampuchea or Cameroon or Cameroons or Cameron or Camerons or Cape Verde or Central African Republic or Chad or China or Colombia or Comoros or Comoro Islands or Comores or Mayotte or Congo or Zaire or Costa Rica or Cote d'Ivoire or Ivory Coast or Cuba or Djibouti or French Somaliland or Dominica or Dominican Republic or East Timor or East Timur or Timor Leste or Ecuador or Egypt or United Arab Republic or El Salvador or Eritrea or Ethiopia or Fiji or Gabon or Gabonese Republic or Gambia or Gaza or Georgia Republic or Georgian Republic or Ghana or Grenada or Guatemala or Guinea or Guiana or Guyana or Haiti or Honduras or India or Maldives or Indonesia or Iran or Iraq or Jamaica or Jordan or Kazakhstan or Kazakh or Kenya or Kiribati or Korea or Kosovo or Kyrgyzstan or Kirghizia or Kyrgyz Republic or Kirghiz or Kirgizstan or Lao PDR or Laos or Lebanon or Lesotho or Basutoland or Liberia or Libya or Macedonia or Madagascar or Malagasy Republic or Malaysia or Malaya or Malay or Sabah or Sarawak or Malawi or Mali or Marshall Islands or Mauritania or Mauritius or Agalega Islands or Mexico or Micronesia or Middle East or Moldova or Moldovia or Moldovian or Mongolia or Montenegro or Morocco or Ifni or Mozambique or Myanmar or Myanma or Burma or Namibia or Nepal or Netherlands Antilles or Nicaragua or Niger or Nigeria or Muscat or Pakistan or Palau or Palestine or Panama or Paraguay or Peru or Philippines or Philipines or Phillipines or Phillippines or Papua New Guinea or Romania or Rumania or Roumania or Rwanda or Ruanda or Saint Lucia or St Lucia or Saint Vincent or St Vincent or Grenadines or Samoa or Samoan Islands or Navigator Island or Navigator Islands or Sao Tome or Senegal or Serbia or Montenegro or Seychelles or Sierra Leone or Sri Lanka or Solomon Islands or Somalia or Sudan or Suriname or Surinam or Swaziland or South Africa or Syria or Tajikistan or Tadzhikistan or Tadjikistan or Tadzhik or Tanzania or Thailand or Togo or Togolese Republic or Tonga or Tunisia or Turkey or Turkmenistan or Turkmen or Uganda or Ukraine or Uzbekistan or Uzbek or Vanuatu or New Hebrides or Venezuela or Vietnam or Viet Nam or West Bank or Yemen or Zambia or Zimbabwe).tw,sh. (1179705)
5. ((developing or less* developed or under developed or underdeveloped or middle income or low* income or underserved or under served or deprived or poor*) adj (countr* or nation? or population? or world or state*)).ti,ab. (78902)
6. ((developing or less* developed or under developed or underdeveloped or middle income or low* income) adj (economy or economies)).ti,ab. (400)
7. (low* adj (gdp or gnp or gross domestic or gross national)).tw. (205)
8. (low adj3 middle adj3 countr*).tw. (8913)
9. (lmic or lmics or third world or lami countr*).tw. (5007)
10. transitional countr*.tw. (139)
11. or/1‐10 (1323187)
12. (sanitation or sewage or sewerage or wastewater or domestic water or (water adj2 (access* or suppl* or quality or quantit* or standard* or drinking)) or hygiene).ti,ab. (161588)
13. Sanitation/(6507)
14. water/or drinking water/or water supply/or water quality/or fresh water/(198561)
15. waste water/or sewage/(35209)
16. or/12‐15 (343517)
17. hand hygiene/or hand disinfection/(5814)
18. (handwash* or soap* or (hand* adj3 (wash* or hygiene or clean*))).ti,ab. (12639)
19. or/17‐18 (14788)
20. respiratory tract infections/or whooping cough/(43029)
21. Sinusitis/(15930)
22. Common Cold/(4075)
23. Otitis Media/(16557)
24. Pharyngitis/(7508)
25. Influenza, Human/(43747)
26. laryngitis/or croup/(3881)
27. Epiglottitis/(947)
28. pneumonia/or bronchopneumonia/or pleuropneumonia/or pneumonia, bacterial/or pneumonia, pneumococcal/or pneumonia, staphylococcal/or pneumonia, viral/(66073)
29. bronchitis/or bronchiolitis/(22955)
30. (ARIs or (respiratory adj (disease* or infection* or illness*)) or sinusitis or common cold* or otitis media or pharyngitis or influenza or flu or coryza or laryngitis or epiglottitis or croup or pneumonia or bronchitis or bronchiolitis or pertussis or whooping cough).ti,ab. (304365)
31. or/20‐30 (385850)
32. 11 and 19 and 31 (205)
33. (diarrh* or dysenter* or gastroenteritis or cholera* or “waterborne infection*” or enterotoxi* or enteric or enteritis or “escherichia coli*” or “e coli” or rotavirus* or mortality or death*).ti,ab. (1570626)
34. diarrhoea/or dysentery/or enteritis/or gastroenteritis/or cholera/or vibrio cholerae/or waterborne diseases/or enterotoxins/or exp escherichia coli/or escherichia coli infections/or rotavirus/or exp mortality/or “causes of death”/or death/or fatal infections/(671938)
35. or/33‐34 (1845583)
36. 11 and 16 and 35 (6244)
37. ((time adj3 (saving* or allocat* or consum* or fetch* or travel*)) or (collect* adj3 (time* or behavio* or chore* or errand* or drudgery or burden* or inconvenien*))).ti,ab. (65859)
38. Time Factors/(1109957)
39. 37 or 38 (1169839)
40. 11 and 16 and 39 (1713)
41. joint diseases/or arthritis/or exp musculoskeletal pain/or back pain/or low back pain/or neck pain/(97943)
42. ((back adj3 (pain* or injur*)) or backpain or ((neck or spine or spinal) adj3 (pain or injur*)) or neckpain or musculoskeletal or (joint adj3 (pain or disease* or injur*)) or arthriti* or osteoarthriti*).ti,ab. (330866)
43. Amenorrhea/or Menstruation/or Menstruation Disturbances/or Menstrual Cycle/or Oligomenorrhea/or Dysmenorrhea/(43803)
44. (menstruat* or menses or menstrual or amenorrh* or dysmenorrh* or oligomenorrh*).ti,ab. (58762)
45. 41 or 42 or 43 or 44 (453245)
46. 11 and 16 and 45 (255)
47. (“quasi experiment*” or quasi‐experiment* or “random* control* trial*” or “random* trial*” or RCT or (random* adj3 allocat*) or matching or “propensity score” or PSM or “regression discontinuity” or “discontinuous design” or RDD or “difference in difference*” or difference‐in‐difference* or “diff in diff” or DID or “case control” or cohort or “propensity weighted” or propensity‐weighted or “interrupted time series” or (before adj5 after) or (pre adj5 post) or ((pretest or pre test) and (posttest or post test)) or “research synthesis” or “scoping review” or “rapid evidence assessment” or “systematic literature review” or “Systematic review” or “Meta‐analy*” or Metaanaly* or “meta analy*” or “Control* evaluation” or “Control treatment” or “instrumental variable*” or heckman or IV or ((quantitative or “comparison group*” or counterfactual or “counter factual” or counter‐factual or experiment*) adj3 (design or study or analysis)) or QED).ti,ab,kw. (3095201)
48. clinical trial/or clinical trial, phase i/or clinical trial, phase ii/or clinical trial, phase iii/or clinical trial, phase iv/or controlled clinical trial/or randomized controlled trial/or pragmatic clinical trial/(784504)
49. controlled clinical trials as topic/or nonrandomized controlled trials as topic/or randomized controlled trials as topic/or pragmatic clinical trials as topic/or case‐control studies/or retrospective studies/or controlled before‐after studies/or interrupted time series analysis/or random allocation/or cohort studies/or follow‐up studies/or longitudinal studies/or prospective studies/or retrospective studies/or propensity score/(2075906)
50. 47 or 48 or 49 (4824377)
51. 32 or 36 or 40 or 46 (8083)
52. 50 and 51 (1912)
53. limit 52 to yr=“2014 ‐Current” (503)

## APPENDIX B RISK OF BIAS TOOL

**Table:**

Question	Coding	Scoring criteria	Decision rules
**1. Confounding**:	Y, PY, PN, N, U		
Was the allocation or identification mechanism able to address confounding	
**Confounding—justification**	Open answer	Justification for coding decision (include a brief summary of justification for rating, mentioning your response to all subquestions, cite relevant pages)	
**Confounding: RCTs**	Y, PY, PN, N, U	**Are criteria adequately addressed?**	– Score “Yes” if all criterion (a)–(f) are satisfied – Score “Probably Yes” if all criteria are satisfied but there is no balance table to justify the random allocation (f) – Score “Probably No” if (f) is not satisfied because there is no balance table reported and there is evidence suggesting a problem in the randomisation, such as baseline coefficients in a diff‐in‐diff regression table are very different or sample size is too small for the procedure used (using stratification when there are less than two units for each intervention and control group in each strata can lead to imbalance) – Score “No” if the sample size is not sufficient or any failure in the allocation mechanism could affect the randomisation process – Score “Unclear” if the paper does not provide details on the randomisation process, or uses a quasirandomisation process for which it is not clear has generated allocations equivalent to randomisation
		a) The authors describe a random component in sequence generation/randomisation method (e.g., lottery, coin toss, random number table).* b) if a special randomisation procedure is used to ensure balance, it is well described (stratification, pairwise matching, unique random draw, multiple random draws etc.) and adjustment is taken into account in the analysis (e.g., stratum fixed effects, pairwise matching variables). c) if the unit of allocation was by beneficiary or group, there was some form of centralised allocation mechanism such as an on‐site computer system to ensure adequate allocation concealment; d) if a public lottery was used for the sequence generation, details were given on the exact settings and participants attending the lottery. e) the unit of allocation is based on a sufficiently large sample size to equate groups on average, and f) a balance table is reported for all subgroups receiving differential treatment within control or treatment groups, comparing means and standard deviations of variables; any unbalanced covariates at individual or cluster level are controlled in adjusted analysis, including cluster‐level variables	
			*If a quasi‐randomised assignment approach is used (e.g., prospective assignment by alternation or alphabetical order), you must be sure that the process truly generates groupings equivalent to random assignment, to score “Yes” on this criteria. In order to assess the validity of the quasirandomisation process, the most important aspect is whether the assignment process might generate a correlation between participation status and other factors (e.g., gender, socioeconomic status, pre‐existing health condition) determining outcomes; you may consider whether assignment is done at cluster level (centralised) and covariate balance is reported in determining this
**Confounding: RDD**	Y, PY, PN, N, U	**Are criteria adequately addressed?**	– Score “Yes” if criteria (a)–(c) are all satisfied including all falsification tests in part (b) – Score “Probably Yes” if participants or practitioners are unblinded but there is low risk of them manipulating the assignment score, and falsification tests suggest no other problems – Score “Probably No” if there are important differences between individuals on both sides of the cut‐off, and any confirmation or falsification tests suggest potential problems. – Score “No” if there are important differences between individuals on both sides of the cut‐off, and all confirmation or falsification tests suggest potential problems – Score “Unclear” if confirmation and falsification tests are not reported
		a) Information about the programme targeting criteria are known, presented in the paper, and used to justify the statistical approach. Demonstration of the relationship between the assignment variable (a continuous variable or a discrete scaled variable with sufficient points either side of the cut‐off) and outcome is done using a graph of the assignment‐outcome relationship. Appropriate functional form may include local linear regression at assignment threshold, or ordered polynomial. The treatment effect may be measured as a change in intercept and/or change in slope (regression‐kink design) b) The relationship between assignment variable and outcomes are unconfounded at the threshold. Support for this can be obtained by confirmation test of no discontinuity at the cut‐off in terms of baseline characteristics around the threshold, and falsification tests such as: – addition of a phase in which intervention is not present, or “placebo time period,” for example, by estimating the pretest relationship between assignment variable and outcomes, as a falsification exercise. Responsiveness of the outcome variable to temporal changes in intervention can also help verify the functional form and to adjust for nonlinearities in the relationship – addition of a nonequivalent outcome, or “placebo outcome.” That is, assessing the effect on a second outcome variable that the intervention should not influence, as a falsification exercise – use of “placebo discontinuity” tests showing no other discontinuities in the assignment variable within the bandwidth of interest, as a falsification exercise 1. Classification of intervention status is not affected by systematic manipulation of the assignment variable by participants or decision makers, as indicated by: – assignment decision rule is concealed from participants and practitioners, or – assignment variable is nonmanipulable by participants, practitioners or other decision makers, or – assignment variable is measured with random error	
		The study should report a histogram of the assignment variable to demonstrate that bunching does not occur around the threshold, and McCrary test should be reported	
**Confounding NRS using statistical matching:**	Open answer	**Are criteria adequately addressed?**	– Score “Yes”, if (a)–(c) (if relevant) are addressed. – Score “Probably yes” if the selection into the programme was done according to clear targeting rules, which are used as matching variables, but there are imbalances remaining after matching – Score “Probably no” if programme assignment was self‐selected by participants and there is no baseline data available to match the participants or groups – Score “No” if matching was done based on variables that are likely to be affected by the programme or any other scenario that affects validity of (a)–(c) – Score “Unclear” if relevant variables are not included in the matching equation, or if matching is based on characteristics collected at endline; or if insufficient details are provided on cluster controls
		a) Information about the programme targeting criteria are known, presented in the paper, and used to justify the statistical approach b) Matching is done on pretest (or time‐invariant) characteristics, including the outcome measured at pretest; matches are geographically local; the variables used to match are relevant (e.g., demographic and socio‐economic factors) to explain both participation and the outcome (so that there can be no evident differences across groups in variables that might explain outcomes); and, for cluster‐assignment, authors control for external cluster‐level factors that might confound the impact of the programme* c) With the exception of Kernel matching, the means of the individual covariates are equated for treatment and comparison groups after matching	
			* Accounting for and matching on all relevant characteristics is usually only feasible when the programme allocation rule is known and there are no errors of targeting. There are different ways in which covariates can be taken into account. Observable differences across groups can be taken into account as covariates in the framework of a regression analysis (e.g., propensity‐weighted least squares) or can be assessed by testing equality of means between groups. Differences in unobservable characteristics can be taken into account using DD, FE, or RE if the only characteristics which are unobserved are time‐invariant
**Confounding: NRS using DD or FE or RE analysis of panel data:***	Y, PY, PN, N, U	**Are criteria adequately addressed?**	– Score “Yes” if (a)–(d) are addressed – Score “Probably yes” selection into the program was done according to clear rules, and equal trends demonstrated, but baseline imbalances between groups remained – Score “Probably no” if equal trends not satisfied, and programme allocation was due to participant self‐selection – Score “No” if equal trends not satisfied, programme allocation was self‐selected by participants, and some relevant time‐varying characteristics are not controlled – Score “Unclear” if insufficient details are provided, for example, on testing the equal trends assumption; or if insufficient details are provided on cluster covariates
		a) Outcomes are measured at pretest (before intervention) and posttest (after intervention) using the same approach b) examination of secular trends in outcomes shows parallel trends across treatment and comparison groups during periods prior to intervention c) the method is combined by well‐conducted statistical matching done according to clear programme allocation rules (see above), and baseline imbalances, including in the outcome are shown to be small d) a comprehensive set of individual time‐varying characteristics is controlled, including any cluster‐level covariates that may affect the impact of the programme (e.g., rainfall)**	
			*DD, FE, and RE regression models are sometimes complemented with matching strategies. This combination approach is superior since it only uses in the estimation the common support region of the sample size, reducing the likelihood of existence of time‐varying unobservable differences across groups affecting outcome of interest and removing biases arising from time‐invariant unobservable characteristics
			** Knowing allocation rules for the programme—or even whether the nonparticipants were individuals that refused to participate in the programme, as opposed to individuals that were not given the opportunity to participate in the programme—can help in the assessment of whether the covariates accounted for in the regression capture all the relevant characteristics that explain differences between treatment and comparison
**Confounding: instrumental variables estimation (IV)**	Y, PY, PN, N, U	**Are criteria adequately addressed?**	– Score “Yes”, if (a)–(d) (if relevant) are addressed – Score “Probably yes” if one of the test required for criterion (b) is not reported but the other is, and the rest of the criterion are addressed and the exogeneity of the instrument is clear – Score “Probably no” if exogeneity of the instrument is not convincing and appropriate tests are not reported – Score “No” otherwise if any of the tests required for criterion (a)–(d) are reported and not satisfied – Score “Unclear” if relevant confounders are controlled for but appropriate statistical tests are not reported; or if insufficient details are provided on cluster controls
		a) an appropriate instrumental variable is used which is exogenously generated: for example, due to a “natural” experiment or random allocation. If the instrument is the random assignment of the treatment, or fuzzy discontinuity, the reviewer should also assess the randomisation procedure or discontinuity assignment, as above b) The joint test for the instruments is significant at the level of *F* ≥ 10, or if an *F* test is not reported, the authors report and assess whether the *R* ^2^ (goodness of fit) of the participation equation is sufficient for appropriate identification; and the identifying instruments are individually significant (*p* ≤ .01) c) the study assesses qualitatively why the instrument only affects the outcome via participation (the exclusion restriction); where at least two instruments are used, the authors report on an over‐identifying test (*p* ≤ .05 is required to reject the null hypothesis); and none of the covariate controls can be affected by participation d) for cluster‐assignment, authors control for external cluster‐level factors that might confound the impact of the programme (e.g., weather, infrastructure, community fixed effects, and so forth) through multivariate analysis	
**Selection bias:** was any differential selection into the study adequately resolved?	Y, PY, PN, N, U	**Are criteria adequately addressed?**	– Score “Yes” if (a)–(d) (if relevant) are satisfied. – Score “Probably yes” if the study used prospective design with adequate concealment, but no (or an incomplete) study flow diagram is reported – Score “Probably no” if there are threats to adequate concealment, for example, individual participants were chosen after cluster assignment was conducted or known, and there are differences between characteristics of the two groups beyond those expected by chance alone – Score “No” if there is evidence of differential recruitment into study arms and differences in characteristics of groups not compatible with chance – Score “Unclear” if it is unclear whether participants could affect their assignment status in response to knowledge of an unconcealed allocation mechanism, and no information is presented about participant characteristics
		a) If the study design is prospective, follow ups are recorded for all eligible participant units from recruitment onwards (i.e., prior to treatment). This is best shown using a study flow diagram, or reporting sufficient information to construct one b) Where the unit of allocation in a prospective study was at group level (geographical/social/cluster unit), allocation was performed on all units at the start of the study, or participants and recruiters are blinded to allocation status c) In retrospective studies (designed after intervention has commenced) and prospective cluster studies where participants are chosen after cluster assignment has been conducted, if those recruiting participants (or those being recruited) are aware of assignment prior to their recruitment and this is unlikely to affect recruitment differentially (e.g., participants chosen randomly using a sampling frame based on census and response rate is high), and the evidence presented does not suggest differences in recruitment across arms or characteristics across individuals (based on balance tests) d) Where evidence suggests there is selection bias into the study due to censoring of data (e.g., immortal time bias), this is accounted for using appropriate statistical methods (e.g., propensity weighted regression, Heckman selection model, proportional hazards model)	
**Selection bias—justification**	Open answer	Justification for coding decision (include a brief summary of justification for rating, mentioning your response to all subquestions, cite relevant pages)	
**Attrition bias:** was any differential selection out of the study adequately resolved?	Y, PY, PN, N, U	**Are criteria adequately addressed?**	– Score “Yes” if (a) overall and (b) differential attrition is negligible, and (c) the reasons for attrition are given and similar in both groups, and (d) respondents were randomly sampled – Score “Probably yes” if overall attrition is <20% and differential attrition <10 percentage points, and respondents were randomly sampled – Score “Probably no” if overall attrition exceeds 20% – Score “No” if overall attrition exceeds 20% and the study does not present evidence attrition is randomly distributed, or there is some indication that the survey respondents were purposively sampled in a way that might have led the sampling to be different between treatment and control groups – Score “Unclear” if no information on attrition is given, or there is attrition but no information provided on the relationship between attrition and treatment status, or there if there is insufficient information on how the sampling was done
		a) Total attrition (losses to follow up) between pre‐ and posttest in the study is <20% b) Differential attrition is <10 percentage points, and whether reasons for attrition are given and similar across groups c) Attrition is sufficiently low and similar reasons for attrition in treatment and control, or the study assesses losses to follow up to be random draws from the sample (e.g., by examining correlation with key characteristics across groups, or an *F* test of attrition on baseline characteristics and interacted with treatment status) d) Study participants are randomly sampled	
**Attrition bias—justification**	Open answer	Justification for coding decision (include a brief summary of justification for rating, mentioning your response to all sub questions, cite relevant pages)	
**Motivation bias: was the process of being observed free from motivation bias?**	Y, PY, PN, N, U	**Are criteria adequately addressed?**	– Score “Yes” if either criterion (a) or (b) are satisfied; – Score “Probably yes” if the study is based on data collected during a trial and there is no obvious issue with the monitoring processes (e.g., due to infrequent monitoring) but authors do not mention potential risks – Score “Probably no” if there was imbalance in the frequency of monitoring in intervention groups, which might have influenced behaviour in treatment and control differentially – Score “No” if authors do not use an appropriate method to prevent Hawthorne and John Henry effects (e.g., blinding of participants) or control them (infrequent measurement, methods to ensure consistent monitoring across groups, measurement using a “pure control”) – Score “Unclear” if it is not clear whether the authors use an appropriate method to prevent Hawthorne and John Henry effects (e.g., blinding of outcomes and, or enumerators, other methods to ensure consistent monitoring across groups)
		a) For data collected in the context of a particular intervention trial (randomised or nonrandomised assignment), the authors state explicitly that the process of monitoring the intervention and outcome measurement is blinded to participants and outcome assessors, or methods are used that would minimise risk of Hawthorne effects, John Henry effects or survey effects such as infrequent observation or outcome questionnaires not referring to the intervention. Authors may adapt also the study design to estimate possible survey and Hawthorne effects (e.g., a “pure control” with no monitoring except baseline end‐line) b) Informed consent is not associated with a particular intervention, as in the case of a regular household survey or a cluster‐RCT, data are collected from administrative records, or in the context of a retrospective (ex post) evaluation	
**Motivation bias—justification**	Open answer	Justification for coding decision (include a brief summary of justification for rating, mentioning your response to all sub questions, cite relevant pages)	
**Deviations from intended interventions:**	Y, PY, PN, N, U	**Are criteria adequately addressed?**	– Score “Yes” if criterion (a)–(e) (if relevant) are satisfied; – Score “Probably yes” if there is no obvious problem but there is no information reported on potential risks related to spillovers or contamination in the control group or if there were issues with spill‐overs but they were controlled for or measured – Score “Probably no” if any of the criterion (a)–(e) are not satisfied but the scale of the issue is not clear – Score “No” if any of the criterion (a)–(e) (if relevant) are not satisfied and happened at a large scale in the study – Score “Unclear” if spill‐overs, no‐shows, cross‐overs, implementation fidelity, or adherence to continuous interventions, are not addressed clearly
		a) There were no implementation issues that might have led the control participants to receive the treatment, or authors use intention‐to‐treat estimation b) The intervention is unlikely to spill‐over to comparisons (e.g., participants and nonparticipants are geographically and/or socially separated from one another and general equilibrium effects are not likely), or the potential effects of spill‐overs were measured (e.g., variation in the % of units within a cluster receiving the treatment) c) There is no risk of substitution (differential contamination) by external programs (also called treatment confounding): participants are isolated from other interventions which might be received differentially between treatment and controls which could explain changes in outcomes d) Errors in implementation fidelity by the intervening body were not systematic, or unlikely to affect the outcome e) For continuous interventions, measurement is taken of adherence to treatment among participants alongside outcomes	
Was the study adequately protected against spill‐overs, no‐shows and cross‐overs?			
**Deviation from intended interventions—justification**	Open answer	Justification for coding decision (include a brief summary of justification for rating, mentioning your response to all sub questions, cite relevant pages)	
**Measurement bias:**	Y, PY, PN, N, U	**Are criteria adequately addressed?**	– Score “Yes” if criterion (a)–(f) are satisfied: – Score “Probably yes” if there is a small risk related to any of (a), (b), (c), (d), (e) of (f) and there is no more information provided to justify the absence of bias, or if potential biases are measured, for example, with placebo outcomes, and found to be null – Score “Probably no” if there clear risks related to (a), (b), (c), (d), (e) or (f) – Score “No” if there clear risks related to (a), (b), (c), (d), (e), or (f) and it is clear that authors were not able to control for the bias – ‐Score “Unclear” if it there is a risk related to any of (a), (b), (c), (d), (e), or (f) and there is no more information provided to justify the absence of bias
Was the study free from biases in measurement of intervention and outcomes?		a) The study is a prospective design or in a retrospective design, participation in the intervention is observed, or the intervention clearly and consistently defined and misreporting by participants or enumerators is unlikely b) Outcomes are clearly and consistently defined for all participants and outcome assessors in the study c) Outcomes are measured through observation (rather than self‐report), and outcome assessors are blinded to intervention or it is shown they are unbiased (e.g., spot‐checks to validate) d) For self‐reported outcomes: respondents in the intervention group are not more likely to have accurate answers than controls due to recall bias e) For self‐reported outcomes: respondents do not have incentives to over/under report something related to their performance or actions, or researchers put in place mechanisms to reduce the risk of reporting bias (outcome assessors not involved in the implementation of the intervention and it is clear that their answers to the survey will not affect what they receive in the future), or authors have measured bias through falsification tests or measuring the effect on placebo outcomes in cases where there was a risk of reporting bias f) Timing of the data collection did not differ between intervention and comparison group, the baseline data are not likely to be differentially affected by the time of intervention (e.g., due to seasonality)	
**Measurement bias—justification**	Open answer	Justification for coding decision (include a brief summary of justification for rating, mentioning your response to all sub questions, cite relevant pages)	
**Analysis reporting bias: RCTs**	Y, PY, PN, N, U	**Are criteria adequately addressed?**	– Score “Yes” if all the criterion (a)–(e) are satisfied – Score “Probably yes” if all the conditions are met except (a), or if all the conditions are met but there is some element missing that could have helped understand the results better (e) – Score “Unclear” if there is not enough information to determine that there is an analysis missing – Score “Probably no” if any of the criterion (b), (c), or (d) are not satisfied; Score “No” if any of the criterion (b), (c), or (d) are not satisfied and there is evidence that the analysis results would be different because large imbalances were not controled for, compliance was very low and ITT estimation was not reported or different treatment arms were pooled
		a) A pre‐analysis plan or trial protocol is published and referred to or the trial was preregistered or the outcomes were pre‐registered b) Authors report results corresponding to the outcomes announced in the method section (there is no outcome reporting bias) c) Authors report results of unadjusted analysis and intention to treat (ITT) estimation, alongside any adjusted and treatment‐on‐the‐treated/complier‐average‐causal‐effects analysis) d) Authors use the appropriate analysis method (use baseline data when available) and different treatment arms are differentiated in the analysis e) Authors have reported all the analysis which could help understand the results and no other bias is assessed as unclear due to the lack of an important analysis (e.g., a balance table or a subgroup analysis)	
Was the study free from selective analysis reporting?			
**Analysis reporting bias: NRS**	Y, PY, PN, N, U	**Are criteria adequately addressed?**	– Score “Yes” if (a)–(d) are satisfied – Score “Probably Yes” if authors combined methods and reported relevant tests (d) only for one method, or if all the criteria are met except for (a) and it is a retrospective NRS – Score “Unclear” if intended outcomes not specified in the paper, or if any of the requirements for d) are not reported – Score “Probably No” if (b) is addressed, but authors did not present results for all outcomes announced in the method section, or did not meet requirement (d) although reported – Score “No” if authors use uncommon or less rigorous estimation methods such as failure to conduct multivariate analysis for outcomes equations, or if some important outcomes are subsequently omitted from the results or the significance and magnitude of important outcomes was not assessed
		a) a pre‐analysis plan is published, especially for prospective NRS (but ideally also for retrospective studies) b) authors use “common” methods of estimation (i.e., credible analysis method to deal with attribution given the data available); c) There is no evidence that outcomes were selectively reported (e.g., results for all relevant outcomes in the methods section are reported in the results section); d) Requirements for specific methods of analysis: – For RDD, Researchers should analyse the change in slope and/or level using different band‐widths around the threshold or functional form. The following should be prespecified as far as possible, and reported in sensitivity analysis: (a) selection of optimal bandwidth using existing data‐driven routines; (b) selection of appropriate functional form for the relationship between assignment and outcome variables; and (c) robustness checks of other bandwidths and functional form specifications – For PSM and covariate matching: (a) Where over 10% of participants fail to be matched, sensitivity analysis is used to re‐estimate results using different matching methods (Kernel Matching techniques); (b) For matching with replacement, no single observation in the control group is matched with a large number of observations in the treatment group, and authors take into account the use of control observations multiple times against the same treatment in the standard error calculation; (c) for PSM, Rosenbaum's test suggests the results are not sensitive to the existence of hidden bias; (d) different matching methods including varying sample sizes yield the same results – For IV models, the authors test and report the results of a Hausman test for exogeneity (*p* ≤ .05 is required to reject the null hypothesis of exogeneity) – ‐ For Heckman selection models, the coefficient of the selectivity correction term (Rho) is significantly different from zero (*p* < .05)	
Was the study free from selective analysis reporting?			
**Analysis reporting bias—justification**	Open answer	Justification for coding decision (include a brief summary of justification for rating, mentioning your response to all sub questions, cite relevant pages)	
**Method used to address differences between UoA and UoR/UoT**	Open answer	Briefly describe methods used to adjust standard errors to account for correlation across units (e.g., cluster‐robust standard errors reported). Unit of analysis (UoA) is the unit of observation (e.g., individual, household, community, village), unit of randomisation (UoR) is the unit of assignment to control or treatment groups (e.g., individual, household, community, village), and unit of treatment (UoT) is the level at which treatment happens (e.g., individual, household, community, village)	
**Unit of analysis: RCTs**	Y, PY, PN, N, U	– Score “Not applicable” if it is there is no clustering in the design at household or group levels – Score “Yes” if UoA equals UoR, or UoA is not equal to UoR and standard errors are clustered at the UoR level, or data is collapsed to the UoR level – Score “Not reported/unclear” if not enough information is provided on the way the standard errors were calculated or what the unit of analysis is. In this case, authors should consider adjusting standard errors using variance inflation formula in sensitivity analysis – Score “No” otherwise. In this case, standard errors should be adjusted using variance inflation formula	
Is unit of analysis in cluster allocation addressed in standard error calculation?			
**Unit of analysis: NRS**	Y, PY, PN, N, U	– Score “Not applicable” if there is no clustering in the design at household or group levels. – Score “Yes” if UoA equals UoT, or if UoA is not equal to UoT and standard errors are clustered at the UoT level, or data are collapsed to the UoT level – Score “Not reported/unclear” if not enough information is provided on the way the standard errors were calculated or what the unit of analysis is – Score “No” otherwise. In this case, standard errors should be adjusted using variance inflation formula	
Are correlations between units addressed in standard error calculation?			
